# Conversion of acetone and mixed ketones to hydrocarbons using HZSM-5 catalyst in the carboxylate platform

**DOI:** 10.1371/journal.pone.0277184

**Published:** 2022-11-21

**Authors:** Sebastian Taco-Vasquez, Mark T. Holtzapple

**Affiliations:** 1 Departamento de Ingeniería Química, Escuela Politécnica Nacional, Quito, Ecuador; 2 Department of Chemical Engineering, Texas A&M University, College Station, Texas, United States of America; West Virginia State University, UNITED STATES

## Abstract

In this study, two different feeds were treated to produce hydrocarbons: (1) reagent-grade acetone, and (2) mixed ketones obtained from lignocellulosic biomass via the carboxylate platform. Acetone and mixed ketones underwent catalytic self-condensation over HZSM-5. For acetone, HZSM-5(80) was used, and the experiments were conducted in two sets: (1) vary temperature (305–415°C) at *P* = 101 kPa (abs) and weight hourly space velocity (WHSV) = 1.3 h^–1^; (2) vary WHSV (1.3–7.9 h^–1^) at *T* = 350 and 415°C, and *P* = 101 kPa (abs). For acetone over HZSM-5(280), the experiments were conducted in two sets: (1) vary WHSV (1.3–6.5 h^–1^) at *T* = 415°C, and *P* = 101 kPa (abs); and (2) vary WHSV (1.3–11.8 h^–1^) at *P* = 790 kPa (abs) and *T* = 415°C. For mixed ketones, HZSM-5(280) was used at WHSV = 1.9 h^–1^, *T* = 430–590°C, and *P* = 101 kPa (abs). For acetone at higher temperatures, the conversion was 100% and the liquid products were aromatics centered on C8. At low temperatures, conversion was less and the carbon liquid distribution was centered on C9 (mainly mesitylene). For mixed ketones, catalyst deactivation was higher causing product concentrations to change over time, and the highest conversion reached was 40%.

## 1. Introduction

To address climate change, carbon-neutral transportation fuels are required. Liquid hydrocarbons are preferred for the following reasons: (1) easily transported and stored, (2) high energy density, (3) relatively safe, and (4) compatible with existing infrastructure. Combustion of hydrocarbons inevitably releases carbon dioxide into the atmosphere; therefore, it is necessary to remove carbon dioxide directly from the atmosphere so the carbon can be recycled. Plants are a practical approach, and the least-expensive and most-abundant plant biomass is lignocellulose. Lignocellulose is the building block of plants and contains cellulose, hemicellulose, and lignin. The amount of lignin varies from every other lignocellulosic plant. Generally, lignocellulose biomass constitutes lignin (*10–25*%), cellulose (40–60%), and hemicelluloses (20–40%) [1]. For instance, hardwood trees have lignin (14–34%), cellulose (31–64%), and hemicellulose (25–40%) [2].

A leading option for converting lignocellulose to liquid hydrocarbon fuels is the carboxylate platform [3–5]. In this approach, biomass is biologically converted to carboxylate salts, which are then chemically converted to liquid hydrocarbons. The MixAlco™ process is a version of the carboxylate platform that uses a consortium of microorganisms to digest nearly all biomass components (except lignin and ash) to carboxylic acids (acetic to octanoic). Using a buffer (e.g., calcium carbonate), these acids are neutralized to their corresponding carboxylate salts, which are subsequently chemically transformed into liquid transportation fuels (e.g., gasoline, jet fuel) [4]. Importantly, the MixAlco^TM^ digesters do not require aseptic operating conditions [3]. In one version of the MixAlco^TM^ process, calcium carboxylates are thermally converted to ketones, which are key intermediates. In another version, the ketones are hydrogenated to secondary alcohols, which adds complexity not only because an extra step is required, but an inexpensive source of carbon-neutral hydrogen must be obtained. The ability to directly convert ketones to hydrocarbons greatly simplifies the process.

Although isopropanol and acetone molecules differ by only two hydrogens, their reaction mechanisms are very different. Alcohols quickly lose their oxygen by dehydrating to water. The remaining olefins are subsequently oligomerized to a mixture of hydrocarbons [4]. In contrast, ketones first form their dimer diketone alcohol and then transform into an oxide which, after several steps, becomes aromatics. The ketone route has more steps with oxygenates as intermediates. Over HZSM-5, the reaction of acetone and mixed ketones is exothermic. The ketones react to form hydrocarbons (aromatics), other oxygenates, and water [6].

According to Chang and Silvestri (1977), with HZSM-5 catalyst, acetone undergoes classic acid-catalyzed condensation to mesitylene (also called *aldol condensation*), which occurs when acetone contacts any acid. For example, when acetone contacts sulfuric acid for a long time, it forms an aldol. If the temperature is high enough, the aldol forms mesitylene [7].

Because zeolites have catalytic acid sites in their structure, the reaction of acetone with sulfuric acid is similar to the reaction of acetone with zeolite; both zeolite (HZSM-5) and acid catalyze the reaction. The product spectrum depends on the experimental conditions (e.g., temperature, pressure, and catalyst). According to Salvapati *et al*. (1989), the catalytic self-condensation of acetone is very complex and has numerous products, including diacetone alcohol, mesityl oxide, phorone, mesitylene, isophorone, and 3,5-xilenol. Over an acid catalyst, the first reaction product of acetone is diacetone alcohol. Then, this ketone alcohol is transformed to mesityl oxide (CH_3_C(O)CH = C(CH_3_)_2_) and water. Next, the mesityl oxide reacts with acetone, forming most of the reaction products of the condensation of acetone, e.g., phorone, isophorone, isobutene, acetic acid, mesytilene, and others [8].

Salvapati *et al*. (1989) show the transformation of mesytil oxide to acetic acid and isobutene. This reaction is important because isobutene is oligomerized to mesitylene. According to Salvapati *et al*. (1989), all the aromatic compounds are formed from mesytilene. The dealkylation reaction of mesytilene produces xylenes, toluene, and benzene, in that order [8].

Chang and Silvestri (1977) pioneered the oligomerization of acetone on HZSM-5 catalyst using a packed-bed reactor for their experiments [7]. They studied temperatures from 250 to 400°C using weight hourly space velocity (WHSV) = 8 h^–1^ at 101 kPa (abs), and showed the product distribution of the acetone reaction where the conversion increased from 3.9% (250°C) to 95.3% (400°C). The amount of isobutene decreased significantly with increased temperature from 83.3% (329°C) to 3.6% (399°C). According to Salvapati *et al*. (1989) [8], this decrease may be attributed to the oligomerization of isobutene into aromatics. It is noteworthy that the most abundant hydrocarbon at high temperatures (399°C) is xylene. It is also notable that among all the reaction liquid products (C6^+^), most are aromatics.

Gayubo *et al*. (2004) also reported the oligomerization of acetone on HZSM-5 [9]. Acetone and water (50% mol or 76% mass of acetone) were used in their experiment in a fixed-bed reactor with temperatures ranging from 250 to 450°C with a temperature ramp of 0.5°C/min. For WHSV = 1.2 h^–1^ and *P* = 101 kPa (abs), they studied the effect of temperature on the product distribution. Their results are more detailed than the results presented by Chang and Silvestri (1977). Profiles of aromatics, C5^+^ olefins, C4^+^ paraffins, ethenes, propenes, *n*-butenes, CO, CO_2_, and water were recorded with changing temperature.

At low temperatures (250 to 300°C), aromatic compounds are the most abundant. However, at higher temperatures, the aromatic concentration decreases and the concentration of C5^+^ olefins and isobutene increases. Gayubo *et al*. (2004) [9] showed the effect of co-feeding the acetone/water mixtures with nitrogen, which inhibited the production of aromatics and C4^+^ paraffins. Nitrogen increased the selectivity of propene and C5^+^ olefins. Also, nitrogen reduced catalyst deactivation because it attenuates coke formation.

Acetone was not the only ketone feedstock oligomerized by zeolite catalyst. Fuhse and Bandermann (1987) published experimental results of 10 different ketones (ranging from C3 to C8) compounds over HZSM-5 [10]. Because water is produced during ketone conversion, atomic oxygen is eliminated. Therefore, the atomic carbon-hydrogen ratio (C/H), which is less than 0.62, indicates ketones were easily converted to hydrocarbons. For example, acetone (C/H ratio = 0.75) conversion was ~50%; whereas, 2-heptanone (C/H ratio = 0.58) conversion was 100% at time on stream (T.O.S.) = 500 min. According to Fuhse and Bandermann (1987), the ketone reaction products were mainly aromatic hydrocarbons, predominantly xylene. The hydrocarbons produced are in the gasoline fraction (C5–C11).

**Fig 1:**
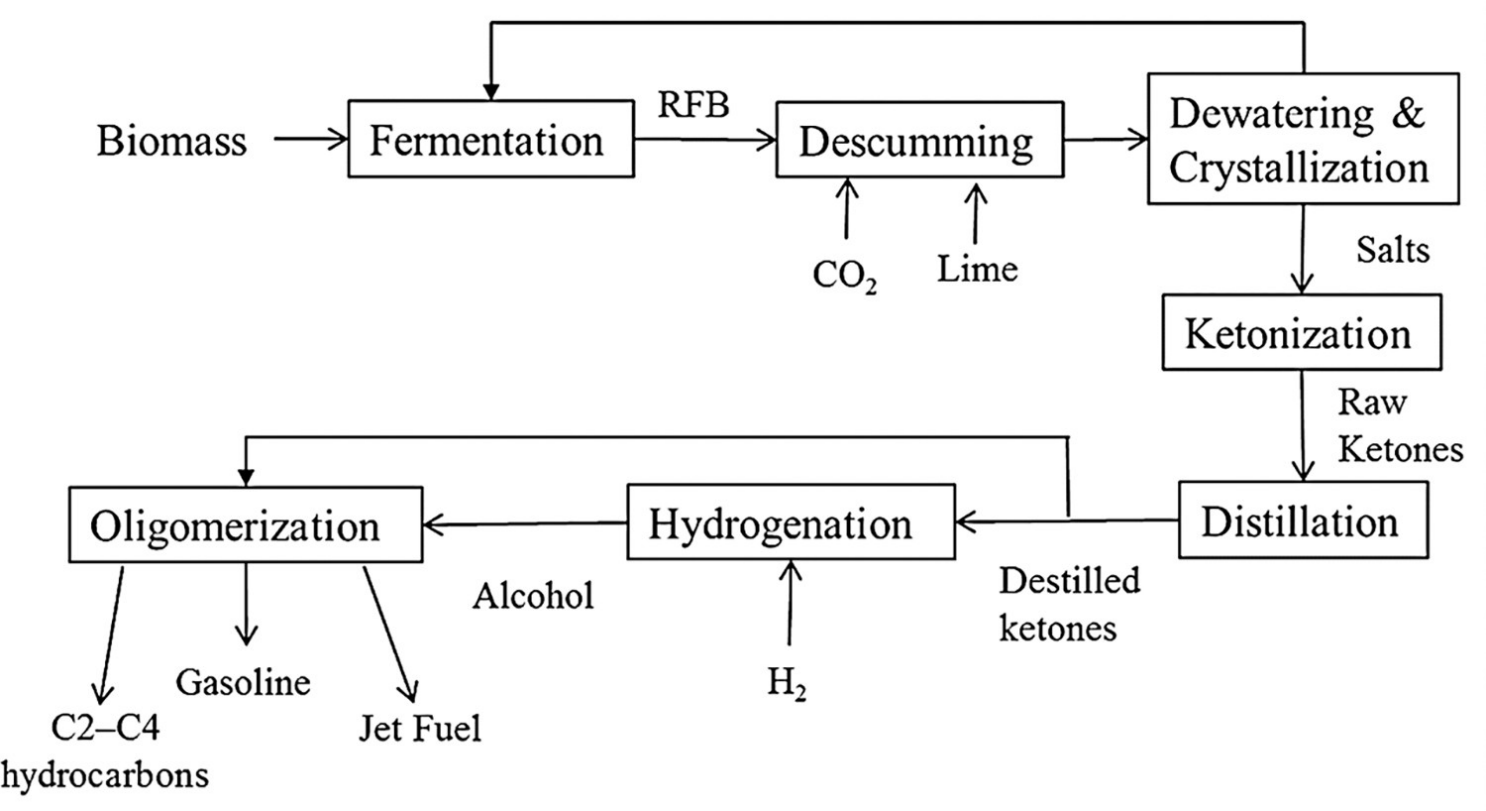
Simplified process block diagram of the MixAlco™ process.

For this study, mixed ketones were produced using the MixAlco^TM^ process (Fig 1). The ketones produced and the C/H ratio of the mixed ketone produced from paper and chicken manure ranged from C3 (C/H ratio = 0.75) to C13 (C/H ratio = 0.54) [4]. According to Fuhse and Bandermann (1987), it is expected that acetone, 2-butanone, and 2-pentanone with C/H ratio > 0.6 to have lower conversion than the remaining ketones (C/H ratio < 0.6) [10].

Tago *et al*. (2011) studied the transformation of acetone to light olefins in a fixed-bed reactor using nano- and macro-crystal size ZSM-5 at atmospheric pressure, *T* = 400°C, and WHSV = 0.5 h^–1^ [11]. For the macro-sized zeolite, the conversion decreased from 65% (T.O.S. = 195 min) to 31.9% (T.O.S. = 385 min) because of the deposition of coke in the acidic sites. In contrast, the nano-sized zeolite exhibited higher conversion decreasing from 98.5% (T.O.S. = 195 min) to 96.5% (T.O.S. = 385 min). Moreover, because olefins reacted with each other to form aromatics at the acid sites located on the outer surface, the nano-sized aromatics yield is 54.3% (T.O.S. = 60 min), which is higher than micro-sized aromatic yield of 27% (T.O.S. = 195 min).

Tago *et al*. (2011) also proposed a possible reaction pathway. First, acetone undergoes aldol condensation where acetone dimerizes to diacetone alcohol, which then dehydrates to mesityl oxide. Then, trimerization occurs producing phorone and iso-phorone, which react to isobutylene, the main intermediate that oligomerizes and cracks to produce light olefins and aromatics [11].

Tago *et al*. (2011) also published experimental results for the transformation of acetone to isobutylene using HBEA zeolite and exchanged with alkali metals (Na-, K-, Rb- and Cs-BEA) at atmospheric pressure, *T* = 500°C, and WHSV = 0.5−0.75 h^–1^. For H-BEA (SiO_2_/Al_2_O_3_ = 13.5), the acetone conversion was high (98%) with T.O.S. = 30 min; whereas, for H-BEA(250) produced a low conversion (63%) at the same reaction conditions. For the BEA zeolites exchanged with alkali metals, it was determined that the order of catalytic activity was high to low according to the following: H-BEA, Na-BEA, K-BEA, Rb-BEA, Cs-BEA. This pattern is similar to the order of their acidity [12].

Cruz-Cabeza *et al*. (2012) studied the transformation of acetone to hydrocarbons in a micro-analytical pulse reactor using 10 mg of zeolite β and helium as carrier gas (100 mL/min) at *T* = 400°C [13]. The metal cation-exchanged zeolite catalysts were prepared from NH4-β(12.5) purchased from Zeolyst International (Pennsylvania, USA), calcined at 600°C for 3 h to obtain its protonated form, and exchanged with metals: Cr, Mn, Fe, Co, Ni, Cu, Zn, Al, Pb. H-β and Al-β showed the highest acetone conversion of 96.8% and 91.6%, respectively; whereas, Fe-β, Co-β, Ni-β, and Pb-β had conversions lower than 50%. Al-β and H-β had the highest aromatic selectivity with 64.5% and 52.5%, respectively. The gaseous paraffin products were propane, isobutane, isobutylene, butene iso-C5, and iso-C6. Furthermore, low acetone conversion causes high isobutene selectivity. For instance, Co-β conversion is low (~39.6%); however, isobutene yield is 23.2%. On the other hand, H-β conversion is high (~96.8%), whereas isobutene yield is 7.5%.

Cruz-Cabeza *et al*. (2012) [13] also proposed a reaction mechanism for transforming acetone to hydrocarbons where acetone first undergoes an aldolization and dehydration to produce mesityl oxide. Then, mesytil oxide reacts with acetone to produce mesitylene, which dealkylates to produce aromatics. Cruz-Cabeza *et al*. (2012) [13] describe the same reaction pathway as Salpavati *et al*. (1989) where mesityl oxide is the main intermediate to produce aromatics. On the other hand, Tago *et al*. (2011) proposed an alternative intermediate where isobutylene is the main precursor for aromatics and light olefins [11,12].

Kikhtyanin *et al*. (2014) studied the aldol condensation of furfural and acetone interaction using zeolites HZSM-5(23), HZSM-S(50), HBEA(25), HBEA(38), HMOR(20), HSDUSY(80), and HSUSY(5) in a 1-L magnetic-stirred glass batch reactor loaded with 39.5 g of acetone and 6.5 g of furfural at *T* = 20, 60, and 100°C with T.O.S. = 0−24 h [14]. The furfural reacts with acetone to produce 4-(2-furyl)-3-buten-2-one (FAc) and water. FAc reacts again with furfural to produce 1,4-pentandien-3-one-1,5-di-2-furanyl (F_2_Ac). Also, FAc can dimerize to produce (FAc)_2_. For HBEA(25) with T.O.S. = 8 h, the furfural conversion increased with temperature from 31% (60°C) to 50% (100°C). Moreover, for HBEA(25) at *T* = 100°C with T.O.S. = 8 h, the selectivities of FAc, F_2_Ac, and (FAc)_2_ were 79, 3.7, and 16.8%, respectively. FAc is more abundant because it is a smaller molecule than F_2_Ac and (FAc)_2_. HBEA has a pore size ~0.67 nm; therefore, larger molecules (F_2_Ac and (FAc)_2_) are impossible products to form inside the pore channels of HBEA.

On the other hand, Kikhtyanin *et al*. (2014) did not report mesityl oxide occurring when two molecules of acetone react to produce 4-methylpent-3-en-2-on (mesityl oxide). Mesityl oxide is the precursor of mesytilene, isophorone, and aromatics, and occurs at higher temperatures (*T* ≥ 305°C); however, Kikhtyanin *et al*. (2014) operated at lower temperatures (*T* ≤ 100°C).

Witsuthammakul and Sooknoi (2015) studied the selective hydrodeoxygenation of acetone, methyl ethyl ketone (MEK), and cyclohexanone to olefins in a fixed-bed reactor with H_2_ flow = 30 mL/min and temperatures ranging from 100 to 300°C [15]. The catalyst beds tested follow: (1) double bed of 5% Ni/SiO_2_ and HZSM-5(13), (2) mixed physical bed of 5% Cu/SiO_2_ with HZSM-5(13), and (3) 5% Cu/HZSM-5(250) or 5% Cu/HY(100) bifunctional catalyst. For Test 1, the acetone and MEK conversions were the same ~60%. For Test 2, the acetone, MEK, and cyclohexanone conversion were 68, 56, and 100%, respectively. Because it has stronger adsorption than acetone or MEK, cyclohexanone is more reactive than acetone and MEK. For Test 3 with Cu/HZSM-5(250), the conversion of acetone increased from 51 to 100% when the space time increased from 19 to 83 g·h/mol. On the other hand, for 5% Cu/HY(100), the acetone, MEK, and cyclohexanone conversions were 91, 25, and 85%, respectively.

Witsuthammakul and Sooknoi (2015) concluded that nickel catalyst had higher conversion than copper, and it promotes hydrogenation of the olefin produced; consequently, a double-bed system containing Ni/SiO_2_ and zeolites is ideal for the hydrodeoxygenation of ketones to olefins. On the other hand, copper can only hydrogenate ketone to alcohol without hydrogenolysis. Zeolites can only dehydrate the alcohol formed and oligomerize it. Nickel catalysts hydrogenation occurs above 100°C, and hydrogenolysis above 175°C. HZSM-5 and HY dehydrate the alcohol product between 150 and 200°C, and oligomerization to olefins occurs at *T* ≥ 200°C. On the other hand, copper catalyst hydrogenates above 100°C, but can only hydrogenate ketone to alcohol, and not olefins to paraffins after oligomerization.

Zhengsheng *et al*. (2022) studied the aldol condensation of aldehydes and ketones followed by hydrogenation to produce aviation-fuel-range hydrocarbons (C9 to C16) [16]. The reactants were furfural, acetone, butanone, and butyraldehyde using tetrahydrofuran as solvent with a bifunctional catalyst of Ni/Mg and Al-O/activated carbon in a batch reactor. The experiments had two stages: (1) first aldol condensation occurred at *T* = 170°C, and *P* = 3000 kPa (abs) for 9 h; (2) then N_2_ and H_2_ were injected and hydrodeoxygenation started at *T* = 260°C, and *P* = 4500 kPa (abs) for 12 h. The aldol condensation products conversion was 71.01%. Acetone or butanone react with the aldehyde to produce dimers or trimers; however, these dimers and trimers still contain double-bond olefins and oxygen in the form of ketone or alcohol radicals. Therefore, a second stage was needed where hydrodeoxygenation saturates the double bonds and eliminates the oxygen as water. The final hydrocarbon products are only paraffins ranging from C7 to C14, with C10 having the highest concentration. Witsuthammakul and Sooknoi (2015) also studied a bifunctional type of catalyst; however, they used a packed-bed reactor. Witsuthammakul and Sooknoi (2015) concluded that the bifunctional catalyst first hydrogenates the ketone to alcohol and then dehydrates and oligomerizes the alcohol. On the other hand, Zhengsheng *et al*. (2022) concluded that aldol condensation of ketones occurs first, followed by the hydrodeoxygenation of dimer or trimer products to paraffins and water in a two-stage process.

The objective of this paper is to transform acetone and mixed ketones to liquid hydrocarbons using HZSM-5 catalyst. For the MixAlco™ process, the direct transformation of mixed ketones into hydrocarbons eliminates their hydrogenation to secondary alcohols, which reduces the number of steps to obtain hydrocarbons (e.g., jet fuel or gasoline). Although mixed alcohols and mixed ketones products have the same carbon distribution, their reaction mechanisms are very different. The purpose of this study is to extend the previous work on mixed alcohols to hydrocarbons [17,18] and investigate the transformation of acetone and mixed ketones to hydrocarbons. This paper also provides an insight into the reaction conditions and the type and distribution of hydrocarbons products obtained, as well as a proposed reaction mechanism. However, the main novelty of this study lies in reporting detailed information about the types of liquid-phase hydrocarbons products from acetone and mixed ketones according to their carbon number and types of hydrocarbons.

## 2. Materials and methods

Reagent-grade acetone (99.5% pure) was obtained from Sigma-Aldrich (Burlington, MA, United States). Mixed ketones were made in the pilot-scale MixAlco™ process located at Texas A&M University. The fermentation was conducted at low temperature, which favors higher molecular weight acids. Taco *et al*. (2014) provide a more detailed description of the steps to obtain mixed ketones [4]. Mixed ketones are yellow with a very strong odor. When oxygen is present, the mixed ketones turn black over time. To avoid color changes, the bottle must be purged with nitrogen to displace air, and then be well-sealed.

GC-MS analysis of the raw mixed ketones mixture showed that it contained only ketones ranging from C3 to C13. After ketonization of the calcium carboxylates produced by the MixAlco^TM^ process, the raw mixed ketones were black; thus, they were distilled to remove the dark color. The dark color was coke from the high-temperature ketonization process. The distilled mixed ketones were a clear yellow color and contained only ketones [2].

Commercial HZSM-5(80) and (280) were purchased from Zeolyst International (Malvern, PA, product # CBV-28014, SiO_2_/Al_2_O_3_ = 280, surface area = 400 m^2^/g 20% alumina binder). The manufacturer supplied cylindrical extruded pellets (diameter = 1.6 mm, length = 3.5 mm), which were packed near the middle section of the reactor. As received, the catalyst was in the ammonium form NH_4_-ZSM-5. To obtain an acid structure, the catalyst was activated with an air stream at 550°C for 3 h, which drove off the ammonia. The weak/strong acidity ratio found in literature (determined by Temperature-Programmed Desorption of Ammonia-NH_3_-TPD) was 0.99 (HZSM-5(80)) and 0.70 (HZSM-5(280)) [19].

### 2.1. Reactor design

**Fig 2:**
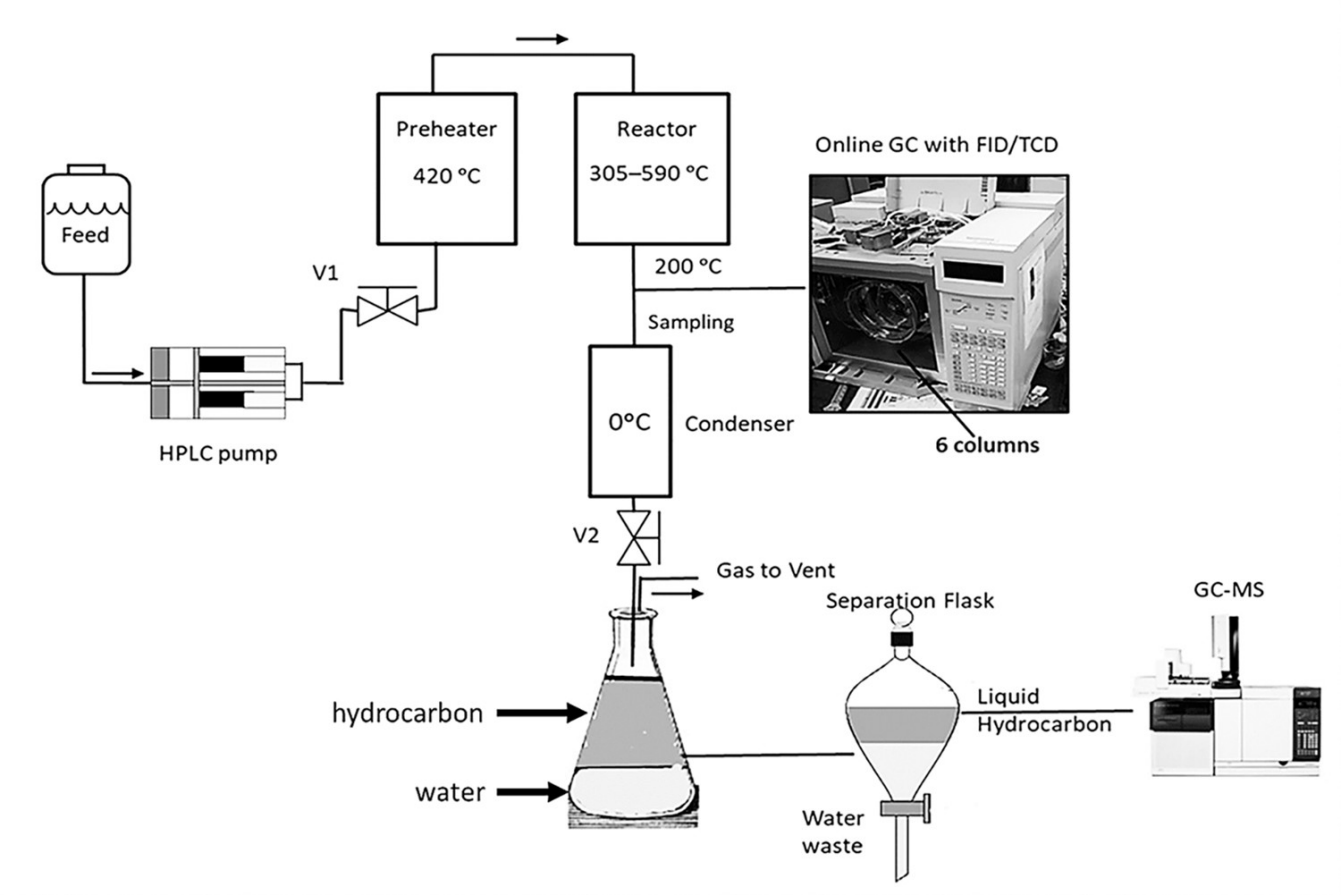
Schematic diagram of the oligomerization apparatus.

The packed-bed reactor was made of stainless-steel tubing with dimensions 10 mm (internal diameter) × 357 mm (length). The top and bottom sections of the reactor were filled with glass beads as an inert packing before and after the catalytic bed. The oligomerization reactor (Fig 2) consists of a packed-bed reactor, a pre-heater, an HPLC pump, mass flow meters, and gas lines for nitrogen and air. Air was used to activate catalyst and for decoking. The reactor, valves, and pipes were constructed of Type-316 stainless steel and were purchased from Swagelok Company (Solon, Ohio, United States). For higher pressure, a dome-loaded back-pressure regulator (Model: S-91KW, *P* range = 3000 psi, *T* range = –65 to 200°C, REDQ Regulator, Salt Lake City, UT, United States) was installed at the reactor exit before the condenser in the oligomerization reactor. The back-pressure regulator was connected to a N_2_ tank at the desired set point pressure. The gas was injected into the dome of the regulator. This pressure seals off the fluid or gas flow from the process. Once the process pressure into the regulator exceeds the dome pressure, the diaphragm flexes and allows the fluids and gas to pass, thus maintaining the process pressure. When the pressure of the process drops below the dome pressure, the diaphragm again seals off the process and maintains the pressure. For these experiments, the set point pressure was 790 kPa in the system.

To vaporize the ketone feed, the pump injected liquid into the preheater, which operated around 420°C. Then, the ketone vapor entered the reactor where it contacted the HZSM-5 catalyst and reacted. Later, the reaction products were heated by heating tape (*T* ~200°C), which ensured that all the products were in the gas phase for the gas chromatograph. Finally, an ice-cooled condenser separated liquid from gas. The gas went to a vent, whereas the liquid was collected for analysis by a gas chromatograph-mass spectrograph (GC-MS).

For the acetone reaction over HZSM-5(80), the oligomerization apparatus reactor depicted in Fig 2 was used. The experiments were conducted in two sets: (1) vary temperature (305 to 415°C) at WHSV = 1.3 h^–1^, and (2) vary WHSV (1.3–7.9 h^–1^) at *T* = 350 and 415°C. The reaction pressure was 101 kPa (abs). For acetone over HZSM-5(280), the experiments were conducted in two sets: (1) vary WHSV (1.3–6.5 h^–1^) at *T* = 415°C, and *P* = 101 kPa (abs); and (2) vary WHSV (1.3–11.8 h^–1^) at *P* = 790 kPa (abs) and *T* = 415°C.

For the transformation of mixed ketones to hydrocarbons, experiments were performed with temperatures ranging from 430 to 590°C. The WHSV studied was 1.9 h^–1^, and the catalyst was HZSM-5(280). For mixed ketones, HZSM-5(280) was chosen because it deactivates slower than HZSM-5(80). WHSV = 1.92 h^–1^ was chosen because a higher WHSV results in more unreacted ketones products. For acetone and mixed ketones, Table 1 shows the number of experiments and their reaction conditions.

**Table 1:** Experiments for acetone and mixed ketones over HZSM-5.

*P*	Catalyst	*T*	Feed	WHSV (h^–1^)							
(kPa)	HZSM-5 (mol silica/mol alumina)	(°C)		1.3	1.9	2.6	3.9	5.2	6.5	7.9	11.8
101	HZSM-5(80)	305	Acetone	E1							
		350	Acetone	E2		E3	E4	E5			
		415	Acetone	E6		E7	E8	E9	E10	E11	
	HZSM-5(280)	415	Acetone	E12		E13	E14	E15	E16		
		430	Mixed Ketone		E22						
		460	Mixed Ketone		E23						
		510	Mixed Ketone		E24						
		560	Mixed Ketone		E25						
		590	Mixed Ketone		E26						
790		415	Acetone	E17			E18	E19		E20	E21

### 2.2. Analytical methods

The reaction products were analyzed by two gas chromatographs: (1) a gas chromatograph (GC) Agilent Technology model # 6890N, and (2) a gas chromatograph-mass spectrometer (GC-MS) HP model # G1800C. The GC was connected on-line with the reactor. This GC had two detectors: (1) flame ionization detector (FID) and (2) thermal conductivity detector (TCD). The TCD analyzed light hydrocarbon products (C1–C4), CO, CO_2_, and water. The FID analyzed heavier hydrocarbons (C5–C13). The GC has six 30-m mega-bore capillary columns: one methyl silicone HP-1, two HP Plot Q, one HP Mole Sieve, one HP Plot Alumina, and one 5% phenyl methyl silicone HP-5 (Agilent Technologies, Santa Barbara, California). All columns have about 40- to 50-μm-thick adsorption phases. The GC has three valves that split the carrier gas into six columns (Fig 2), which better separate the samples and consequently give more accurate results. All the reaction products were analyzed with this chromatograph; however, heavier hydrocarbons (C5–C13) were lumped by carbon number. To identify all the isomers in the liquid phase, the GC-MS analyzed the liquid product samples. Before the analysis, all reaction products were cooled to 0°C to ensure that all C5+ hydrocarbons were in the liquid phase. A GC-MS analysis of the liquid phase typically determined that the liquid samples had over 100 compounds.

## 3. Results

### 3.1. Acetone conversion

#### 3.1.1. Catalyst stability

**Fig 3:**
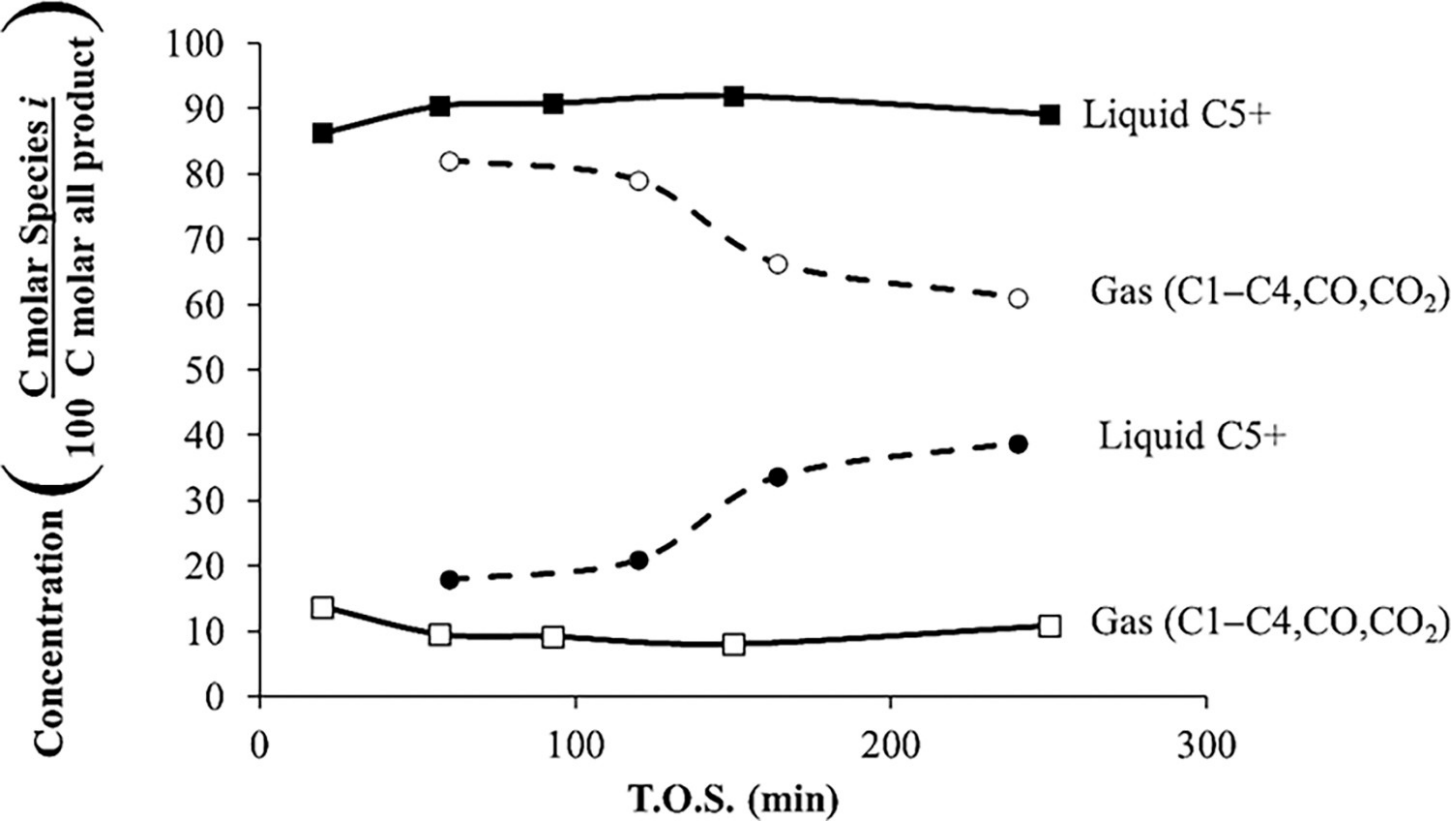
Product distribution of gases and liquids for the acetone reaction over HZSM-5(80) (dashed line) and HZSM-5(280) (solid line), *T* = 415°C, WHSV = 1.3 h^–1^, and *P* = 101 kPa (abs).

Fig 3 shows the percentage of liquid and gas with respect to T.O.S. for acetone over HZSM-5(80) at *T* = 415°C, WHSV = 1.3 h^–1^, and *P* = 101 kPa (abs). The conversion was 100% at all times. The gas phase contains hydrocarbons from C1 to C4, CO_2_, and CO, and the liquid phase contains hydrocarbon C5^+^ (mainly aromatics). Fig 3 shows that with time, the yield for gaseous products decreases and the yield for liquid products increases; therefore, the product selectivity changes with time, which is attributed to catalyst deactivation. On the other hand, Fig 3 also shows the product distribution of liquid and gas phases with respect to T.O.S. for acetone over HZSM-5(280) for *T* = 415°C, *P* = 101 kPa (abs), and WHSV = 1.3 h^–1^. The yields for gaseous products and liquid hydrocarbons did not change with the first 250 min; thus, the deactivation of HZSM-5(280) is slower than HZSM-5(80). HZSM-5(280) is less acidic because it has fewer aluminum atoms, which are bonded to hydrogen or silica proton, and therefore is slow to deactivate compared to HZSM-5(80). For this set of experiments, the conversion is 100%. It is noteworthy that HZSM-5(280) produces 10% of gases, which is much less than the 80% of gases produced with HZSM-5(80). For HZSM-5(280), the amount of gases and liquids are constant during time; therefore, there is low catalyst deactivation with T.O.S. For the acetone reaction over HZSM-5(280), the amount of gaseous products is less abundant than the liquid products. At high temperatures (415°C) and low WHSV (1.3 h^–1^) the acetone conversion is 100%. Fig 3 shows the product distribution of gases and liquids for the acetone reaction over HZSM-5(80) (dashed line) and HZSM-5(280) (solid line) at *T* = 415°C, WHSV = 1.3 h^–1^, and *P* = 101 kPa (abs).

**Fig 4:**
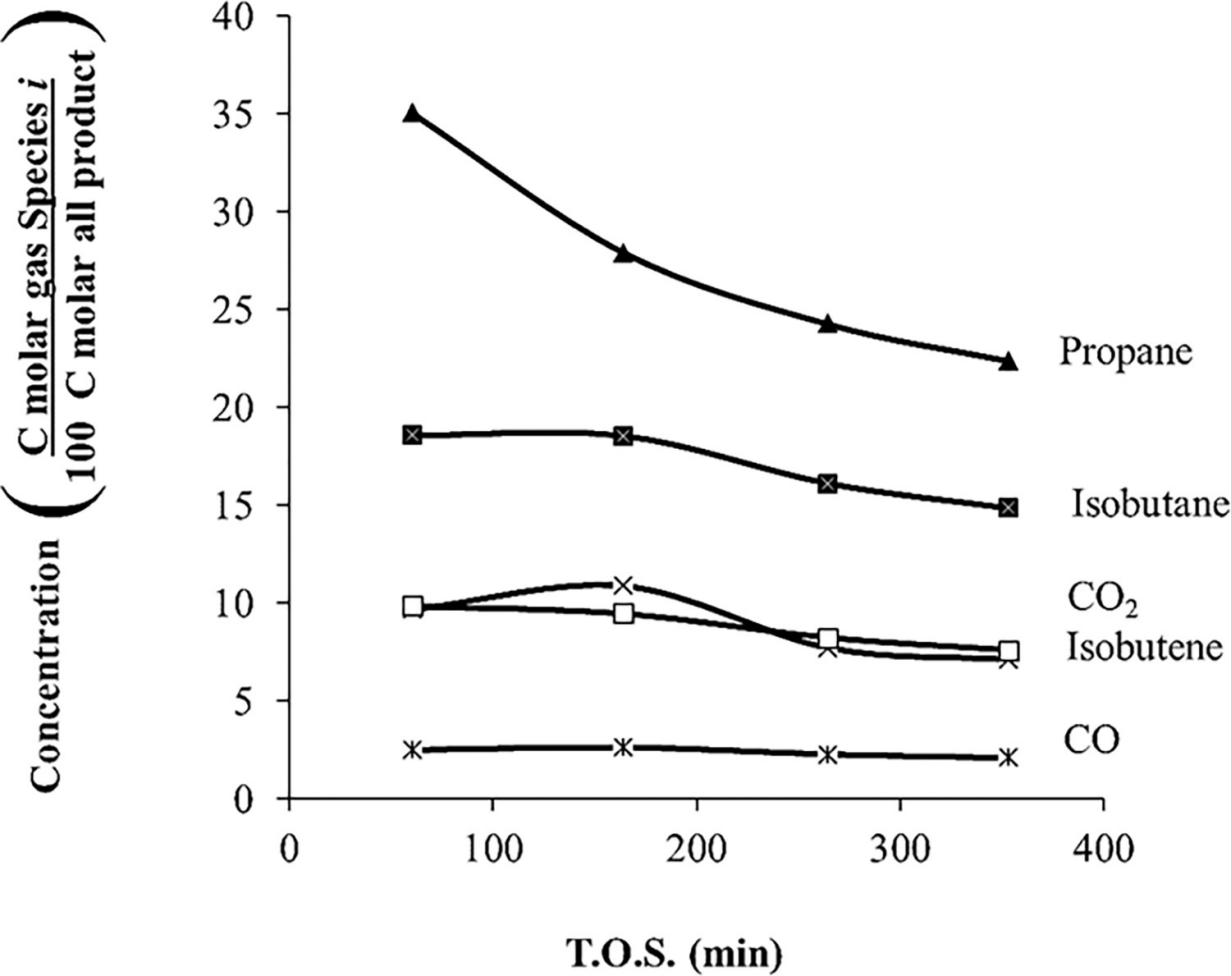
Product distribution of gases for the acetone reaction over HZSM-5(80), *T* = 415°C, WHSV = 1.3 h^–1^, and *P* = 101 kPa (abs).

Fig 4 shows the gas-phase product distribution with respect to T.O.S. for acetone over HZSM-5(80) at the same conditions as the above experiment. Only gases with concentrations over 5 mol% are reported. The most abundant gases are propane and isobutane. For all the gaseous products, the tendency is to decrease with time. For the acetone reaction using HZSM-5(80), temperatures above 400°C are needed to get 100% conversion. At high temperatures, gaseous products are more abundant using HZSM-5(80).

**Fig 5:**
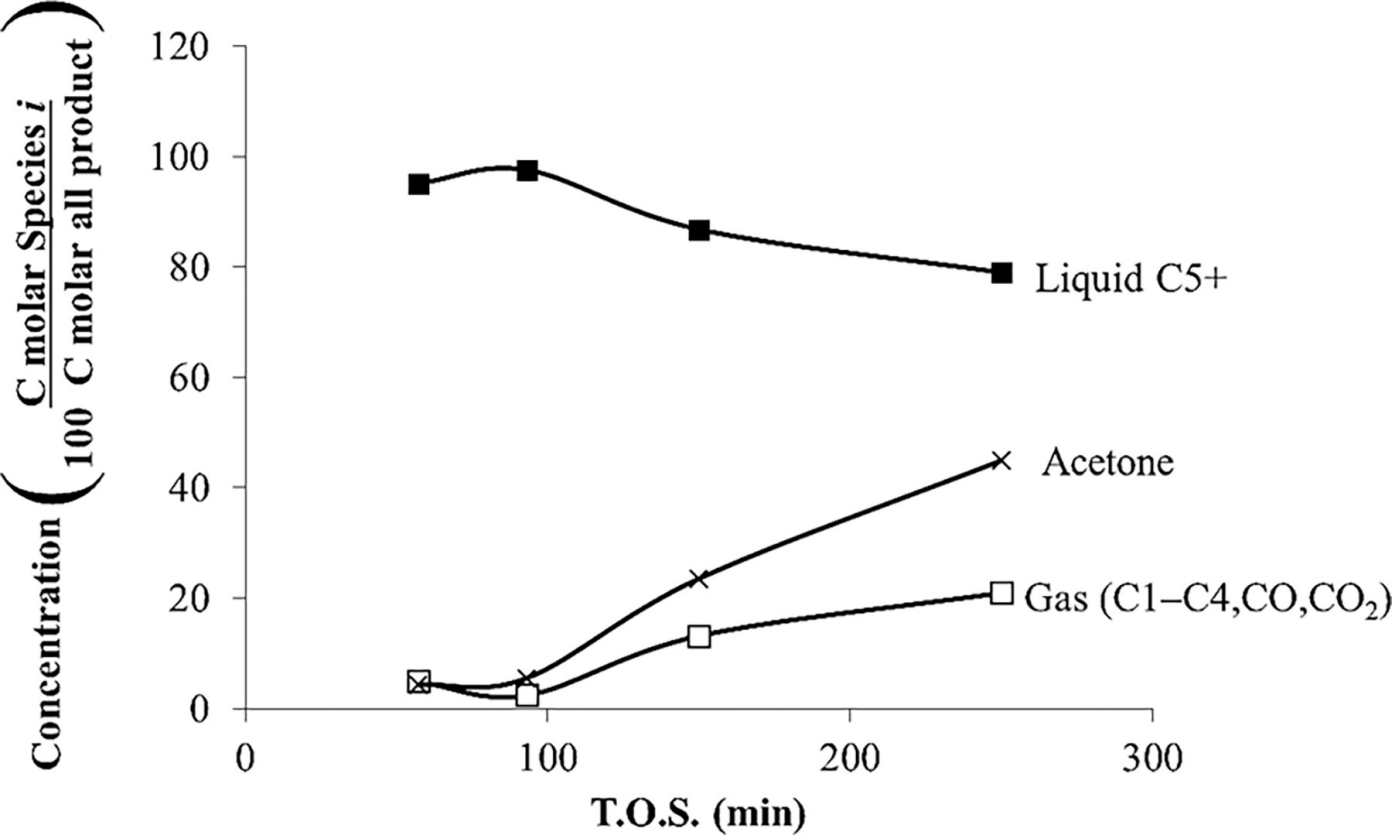
Product distribution of gases and liquids for the acetone reaction over HZSM-5(280), *T* = 415°C, WHSV = 9.48 h^–1^, *P* = 790 kPa (abs).

Fig 5 shows the effect of high pressure for the reaction of acetone over HZSM-5(280) on the conversion and the composition of gaseous and liquid reaction products (*P* = 790 kPa (abs), *T* = 415°C, and WHSV = 9.48 h^–1^). High pressure rapidly deactivates the catalyst. For instance, in Fig 5 the unconverted acetone increased from 5% to 40% after 250 min, which may result from large molecules poisoning the catalyst.

#### 3.1.2. Effect of varying temperature

**Fig 6:**
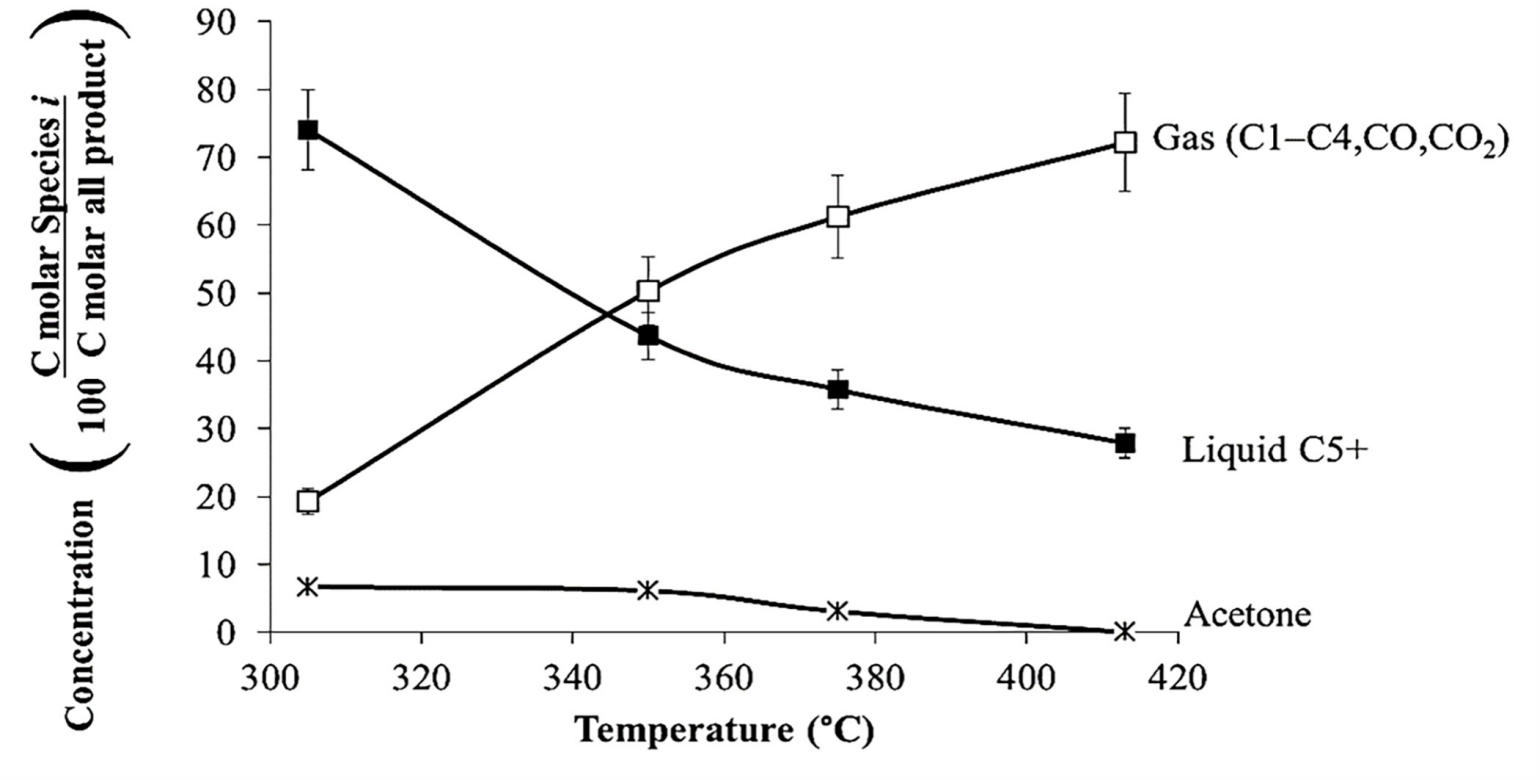
Product distribution of gases and liquids for the acetone reaction over HZSM-5(80), WHSV = 1.3 h^–1^, and *P* = 101 kPa (abs).

Using catalyst HZSM-5(80), Fig 6 shows the distribution of gases and liquid, and the conversion of acetone for *T* = 305 to 415°C, *P* = 101 kPa (abs), and WHSV = 1.3 h^–1^. The amount of gases increased from 20% (305°C) to 72% (415°C); whereas, the amount of liquids decreased from 80% (305°C) to 28% at (415°C). The acetone conversion slightly increased from 90% to 100%.

**Fig 7:**
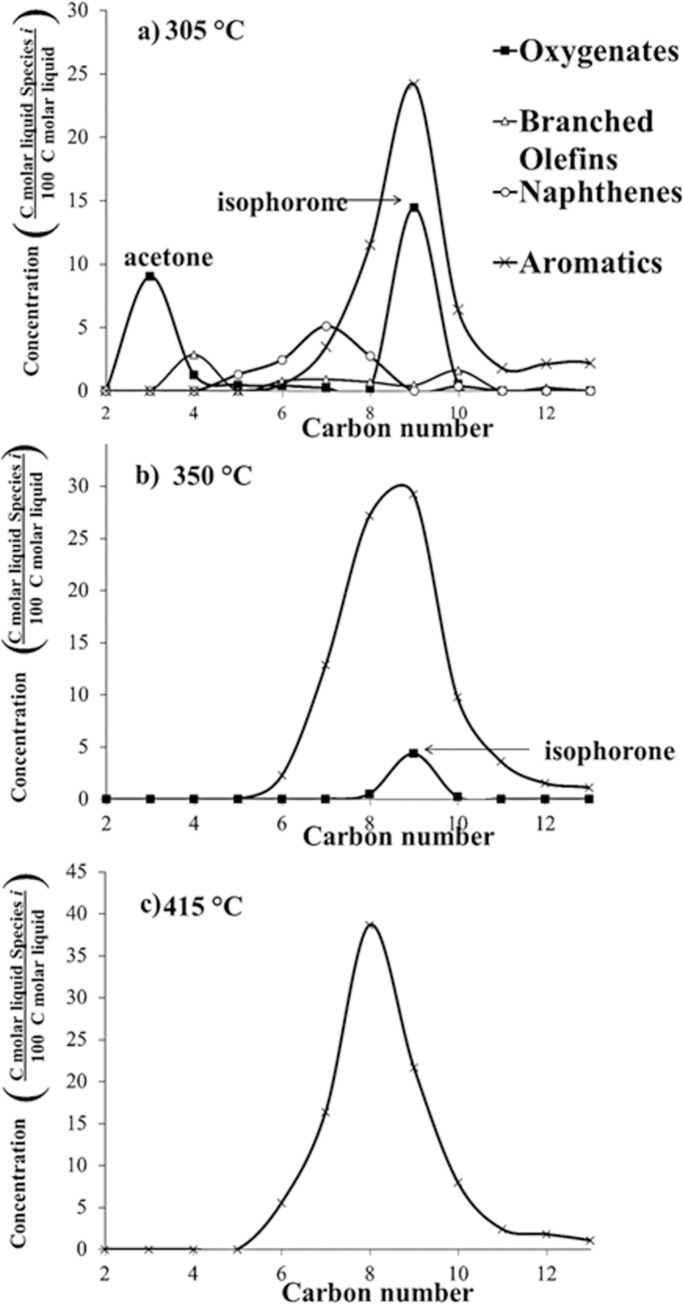
Liquid-type product distribution of acetone reaction over HZSM-5(80), *T* = 305, 350 and 415°C, WHSV = 1.3 h^–1^, and *P* = 101 kPa (abs).

Fig 7 shows the type of liquid-phase products at *T* = 305, 350, and 415°C at *P* = 101 kPa (abs). Aromatics, naphthenes, and oxygenates were the only types of products in the liquid phase. At *T* = 305°C, the most abundant component in the liquid phase was C9, mainly mesytilene (1,3,5-trimethylbenzene, C_9_H_12_) and isophorone (1,1,3-trimethyl-3-cyclohexene-5-one, C_9_H_14_O). The concentration of isophorone decreased from 15 to 0% when *T* increased from 305 to 415°C and the concentration of C9 aromatics decreased from 25% (305°C) to 20% (415°C).

On the other hand, when *T* increased from 305 to 415°C, the concentration of C8 aromatics increases from 15% (305°C) to 40% (415°C), which is attributed to cracking of mesitylene (C9) into xylenes. Wang *et al*. (2008) reported the cracking of mesitylene over HZSM-5 at 480°C and showed that the most abundant reaction product is xylene [20]. According to Wang *et al*. (2008), cracking benefits from increased temperature. For the three experiments at different temperatures, the amount of benzene is less than 5% of the liquid.

Furthermore, it is noteworthy that there is a Gaussian normal distribution of compounds centered on C9 (305°C) and C8 (415°C). The mean can be interpreted as the average carbon number (ACN). The ACN was 8.43±1.4 (305°C) and 8.24 ±1.2 (415°C). This Gaussian distribution of products was not observed in the isopropanol and mixed-alcohol reactions [17,18]. Fig 7 also shows the most abundant compound for each carbon number. For example, at 305°C and C9 fraction, the most abundant aromatic component is mesitylene; whereas at 415°C and C8 fraction, the most abundant aromatic compound is *p*-xylene.

At *T* = 305°C, the amount of naphthenes is less than 5% and ranges from C5 to C9; however, at high temperatures, the amount of naphthenes is about 0%. Table 2 shows the liquid composition at three temperatures: 305, 350, and 415°C. There were about 100 components for each sample; however, Table 2 presents only the most abundant compounds. The total amount of all components for each table represents about 80% (mol) of the total amount of liquid products. The other components that represent 20% (mol) are not shown in the tables because they are numerous, and the concentration is less than 1% (mol).

**Table 2:** Most abundant compound distribution for the acetone reaction over HZSM-5 (80); *T* = 305, 350, and 415°C; WHSV = 1.3 h^–1^; and *P* = 101 kPa (abs) and most abundant compound distribution for the acetone reaction over HZSM-5 (280), *T* = 415°C; WHSV = 1.3, 3.9 and 5.2 h^–1^; and *P* = 790 kPa (abs).

	Composition						
	(C molar liquid Species *i* /100 C molar liquid)						
	T (°C)				WHSV (h^–1^)		
	305	350	415		1.3	3.9	5.2
Aromatics				Aromatics			
Xylenes (orto, meta, para)	10.5	18	30.3	Xylenes (orto, meta, para)	25.6	20.8	13.12
Mesitylene	15	14.8	18.1	Mesitylene	26.29	16.1	16.36
Benzene, 1-ethyl-2-methyl	8.2	12.1	<1	Benzene, 1-ethyl-2-methyl	1.38	10.25	10.06
Toluene	3.4	12.3	16.3	Toluene	13.82	10.3	5.16
Benzene, 1-methyl-3-propyl	1.6	<1	<1	Benzene, 1-methyl-3-(1-methylethyl)	<1	1.24	2.32
Benzene, 1,2-diethyl	1.5	6.6	<1	Benzene, 1,2-diethyl	<1	1.2	1.89
Benzene, 1,2,3,5-tetramethyl	2.1	1.1	<1	Benzene, 1,2,3,5-tetramethyl	5.32	2.57	3.23
Benzene, ethyl	0.9	3	<1	Benzene, ethyl	1.24	2.97	1.47
Napthalenes (all)	0.8	2.5	1.5	Naphthalene, 1-methyl-	1.15	<1	<1
Benzene	<1	1.7	5.5	Benzene	3.11	1.41	1.09
Benzene, 2-ethyl-1,3-dimethyl	<1	2.8	<1	Benzene, 1-methyl-3-propyl-	<1	<1	1.29
Indenes	<1	1.2	<1	1H-Indene, 2,3-dihydro-5-methyl-	<1	1.13	<1
Oxygenated				Oxygenated			
Isophorone	14.1	10.5	<1	Isophorone	5.41	3.9	4.84
Acetone	9	0	0	2-propanone	3.97	4.46	4.32
2-butanone	1.2	0	0	2-butanone	<1	<1	1.44
Others				Others			
Cyclopropane, (1-methylethenyl) C5	2.4	<1	<1	Cyclobutane, isopropyliden-	<1	<1	2.25
Cyclobutane, isopropylidene C7	2.2	<1	<1	1,3-Cyclohexadiene, 1,2,6,6-tetrame	<1	<1	1.48
Cyclopropane, 1,2-dimethyl C5	1.3	<1	<1	Isoterpinolene	<1	<1	1.35
Cyclopentene, 1,5-dimethyl C7	2.1	<1	<1	Cyclopentene, 1,5-dimethyl	<1	<1	1.53
1-Propene, 2-methyl	2.8	1.5	<1	1-Propene, 2-methyl	2.42	2.07	5.94

Table 2 shows that in addition to aromatics and oxygenates, naphthenes are common products. Most of these naphthenes have a double bond in their structure such as cyclopropane (1-methylethenyl). The double bond occurs because of the lack of hydrogen in the acetone reaction to saturate the molecules. Naphthenes can only occur from C3; however, branched naphthenes are most abundant ranging from C5 to C7. Because of the lack of hydrogen in the reaction, naphthenes, aromatics, and olefins are favored over paraffins and isoparaffins.

At low WHSV and high temperatures, the aromatic product distribution is C8-centered. On the other hand, at high WHSV and lower temperatures, the product distribution is C9-centered. The most abundant C9 aromatic compound is mesytilene; whereas, the most abundant C8 aromatics compound is xylene. The amount of isophorone increases at low temperatures and high WHSV. Isophorone is an intermediate that produces aromatics always present in the experiments. For instance, Fig 7 and Table 2 may provide insights into the acetone reaction mechanism over HZSM-5(80) at *T* = 305, 350, and 415°C.

For acetone reaction at low temperatures, naphthenes are reaction products; however, at high temperatures, naphthenes dehydrogenate to produce only aromatics. According to Salvapati *et al*. (1989), Tago *et al*. (2011), and Cruz-Cabeza *et al*. (2012), acetone undergoes aldol condensation to diacetone alcohol, which produces mesityl oxide [8,11–13]. According to Table 2, diacetone alcohol and mesytil oxide are not products found in these experiments; however, they probably are intermediates that appear instantaneously and then are transformed to isphorone and mesitylene, which are among the products in this current paper. According to the literature, another route is isobutylene as the main intermediate that produces hydrocarbons; however, Fig 4 shows that isobutylene has a low concentration compared to other gases. If enough high-temperature energy is supplied, aromatics are cracked and converted to gaseous products (Fig 6).

#### 3.1.3. Effect of varying WHSV

**Fig 8:**
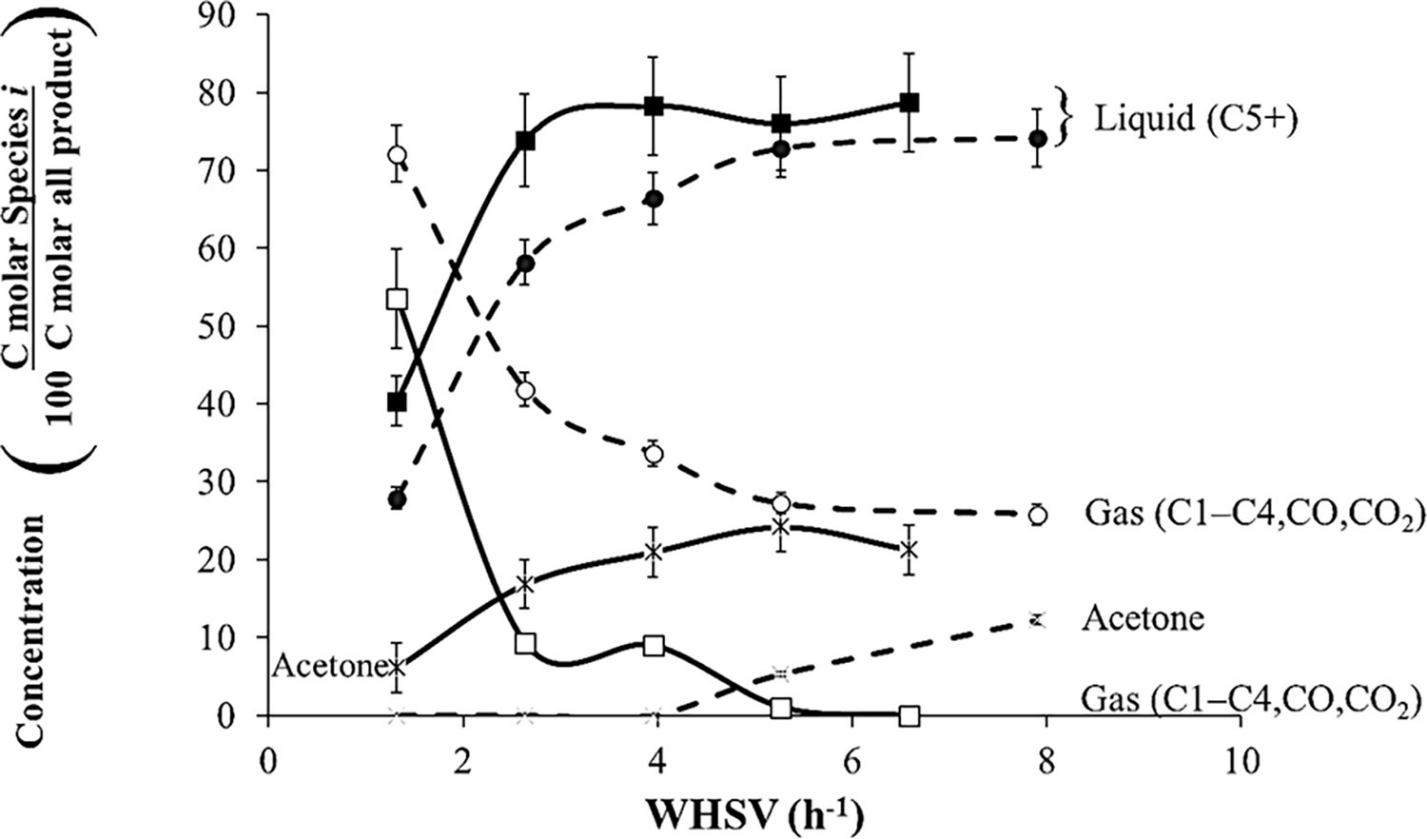
Product distribution of gases and liquids for the acetone reaction over HZSM-5 (80), *T* = 350 (solid line) and 415°C (dashed line), and *P* = 101 kPa (abs).

For HZSM-5(80), *T* = 415°C, and *P* = 101 kPa (abs), Fig 8 shows the acetone conversion at different WHSV (1.32, 2.63, 3.95, 5.27, 6.58, and 7.9 h^–1^). As expected, unreacted acetone is 0% at low WHSV, increasing from 0% (1.32 h^–1^) to 13% (7.9 h^–1^). The amount of gas decreases because there is not enough residence time for oligomerization at high WHSV. The tendency is for all gaseous products to decrease at high WHSV. Fig 8 also shows the distribution of gases and liquid and the conversion of acetone at *T* = 350°C and *P* = 101 kPa (abs) for 1.32, 2.63, 3.95, 5.27, and 6.58 h^–1^ using catalyst HZSM-5(80). The amount of gases decreased from 55% (1.32 h^–1^) to 0% (6.58 h^–1^); whereas, the amount of liquids increased from 10% (1.32 h^–1^) to 80% at (6.58 h^–1^). The unreacted acetone slightly increased from 5% to 23%.

**Fig 9:**
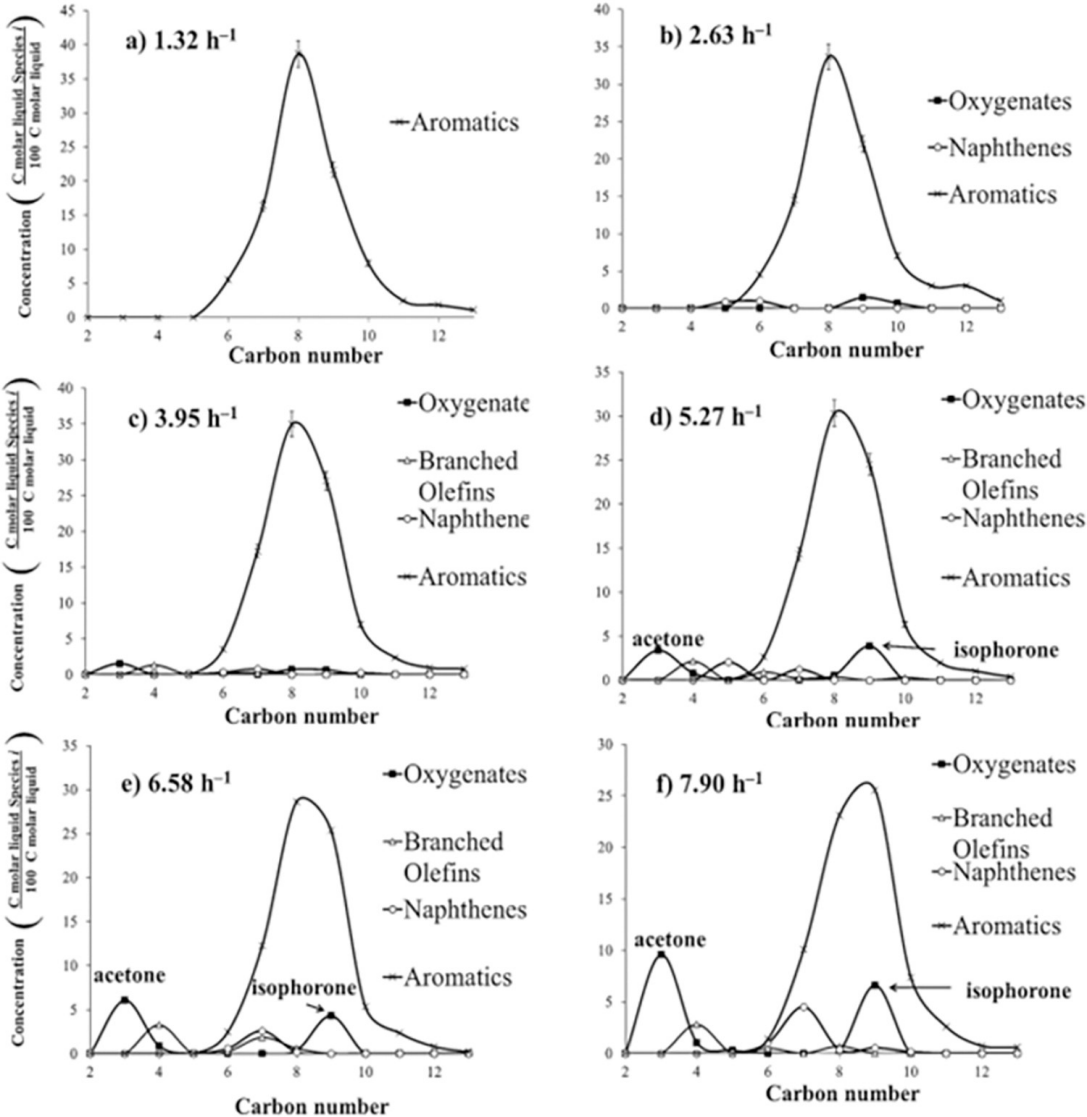
Liquid carbon product distribution of acetone reaction over HZSM-5(80), *T* = 415°C, and *P* = 101 kPa (abs).

At *T* = 415°C and *P* = 101 kPa (abs), Fig 9 shows the type of liquid-phase products for WHSV = 1.32, 2.63, 3.95, 5.27, 6.58, and 7.9 h^–1^. At WHSV = 1.32 h^–1^, the most abundant component in the liquid phase was C8, mainly *p*-xylene. The concentration of isophorone increases from 0 to 7% when WHSV increased from 1.32 to 7.90 h^–1^, and the concentration of unreacted acetone increases from 0% (1.32 h^–1^) to 10% (7.90 h^–1^). When WHSV increases, unreacted acetone increases because there is not enough time for all the acetone to react; therefore, acetone is part of the products. The amount of isophorone increases at low temperatures and high WHSV. Isophorone is an intermediate that produces aromatics and is part of the products when there is not enough residence time to continue reacting.

On the other hand, the concentration of C8 aromatics decreases from 40% (1.32 h^–1^) to 23% (7.90 h^–1^), which is attributed to cracking of mesitylene (C9) into xylene. At WHSV = 1.32 h^–1^, the carbon product distribution is centered on C8; whereas at WHSV = 7.90 h^–1^, the carbon distribution product is centered on C9. For these experiments at different temperatures, the amount of benzene is less than 5% of the liquid. Naphthenes increases from 0 to 5% when WHSV increased from 1.32 to 7.90 h^–1^. Overall, WHSV determines the carbon distribution.

Aromatics have a Gaussian product distribution centered either on C8 or C9, similar to HZSM-5(80). At low WHSV, the aromatic product distribution is C8-centered; however, at high WHSV the product distribution is C9-centered. The amount of isophorone increases at high WHSV. This concurs with the results found by Salvapati *et al*. (1989) where isophorone is an intermediate that at high WHSV does not have time to continue reacting to aromatics.

**Fig 10:**
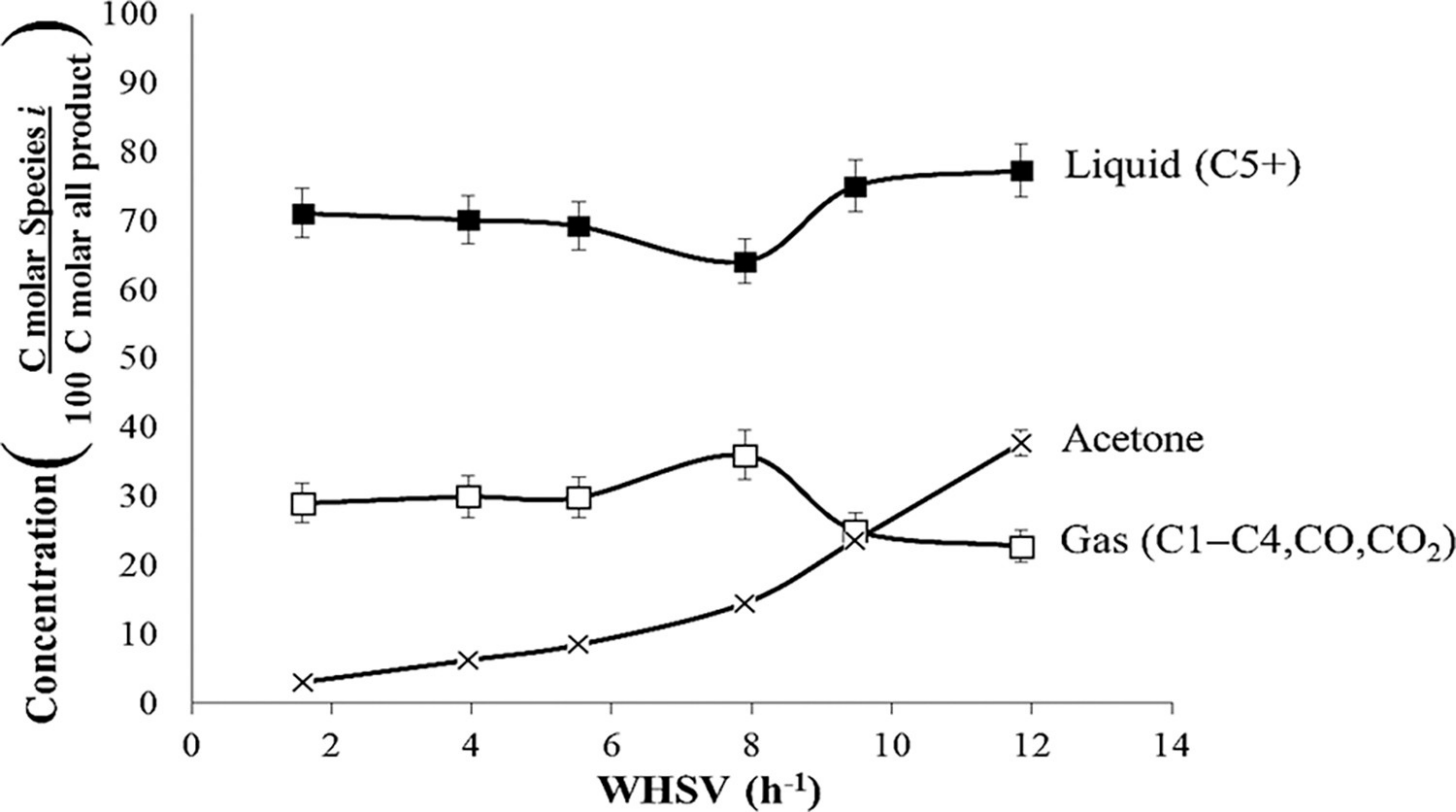
Product distribution of gases and liquids for the acetone reaction over HZSM-5(280), *T* = 415°C, *P* = 790 kPa (abs).

Fig 10 shows the distribution of gases and liquid and the conversion of acetone at different WHSV (1.3, 3.2, 5.5, 7.9, 9.5, and 11.9 h^–1^) using HZSM-5(280) at *T* = 415°C and *P* = 790 kPa (abs). As expected, the acetone conversion is lower at high WHSV. High pressure does not favor the reaction; the high-pressure conversion (Fig 10) is lower than the atmospheric-pressure conversion (Fig 8). Unreacted acetone increased from 3% (1.6 h^–1^) to 37% (11.9 h^–1^). Similar to Fig 8, the amount of liquids is higher than the gaseous products, which uses the same HZSM-5(280).

For the acetone reaction, the effect of increasing the pressure is similar to increasing WHSV or decreasing temperature. Table 2 shows the most abundant compounds at *P* = 790 kPa over HZSM-5(280), and WHSV = 1.3, 3.9, and 5.2 h^–1^. The total amount of all components for each table represents about 80% (mol) of the total amount of liquid products. The other components that represent 20% (mol) are not shown. For instance, the amount of xylenes decreases with WHSV from 25.6% (1.32 h^–1^) to 13% (5.2 h^–1^) at *P* = 790 kPa; whereas, xylenes increase with temperature from 10.5% (305°C) to 30.3% (415°C) at *P* = 101 kPa. Table 2 show the molecular products at *P* = 101 and 790 kPa, respectively, and for both operating pressures most of the compounds are the same; however, catalyst deactivation was higher at high pressure. For instance, unreacted acetone decreases from 4% (*P* = 790 kPa) to 0% (*P* = 101 kPa) at *T* = 415°C; therefore, the catalyst at high pressure is mainly deactivated by coke formation [21] and not by high-molecular-weight compounds blocking the active sites of the catalyst. For acetone reaction over HZSM-5(280) and high pressure (790 kPa), acetone did not reach 100% conversion. Table 2 shows the liquid-product carbon distribution at all WHSV was C9-centered. The amount of liquid product was more abundant than gaseous products. The acetone conversion decreases at high WHSV. High pressure does not favor the reaction. The findings reported herein suggest that high pressure blocks even more of the catalyst pores with coke, thus reducing its activity. This agrees with Guisnet *et al*. (1989) indicating that deactivation occurs through the following three modes: (1) limiting access of feed (acetone or mixed ketone) to the active sites, (2) blocking access to the sites of the cavities (or channel intersections) in which the coke molecules are situated, and (3) blocking access to the pore sites in which there are no coke molecules. For a large part, the pore structure of zeolites determines the deactivating effect of coke. Zeolite HZSM-5 pore system consists of interconnecting channels without cavities. HZSM-5 deactivation occurs initially by limiting access to the active sites, then blocking access to the sites of the channel intersection in which the coke molecules are situated [22]. Lastly, at high coke contents, coke molecules located on the outer surface of the crystallites can block access to the sites of channel intersections. HZSM-5 coking has a moderate deactivating effect compared to beta zeolites [23].

In addition, Figs 9 and 10 also show that low WHSV is equivalent to high temperature where aromatics are the only product; however, high WHSV is equivalent to low temperature where isophorone and mesitylene are the main products.

Table 3 shows the total product mole balance at different temperatures including water and total unreacted acetone for HZSM-5(80), WHSV = 1.3 h^–1^, and *P* = 101 kPa (abs). For acetone reaction using HZSM-5, oxygen is eliminated as CO, CO_2_, or water, and it highly depends on the temperature and WHSV. For instance, at lower temperatures (305°C), water is about 26% whereas CO and CO_2_ were about 0.46 and 1.81%, respectively. On the other hand, at higher *T* = 415°C, water decreases to 11.98% whereas CO and CO_2_ increase to 3.59 and 21.27%, respectively. High temperature leads to more gaseous products.

**Table 3:** Total product distribution for gases and liquids for acetone reaction over HZSM-5(80), *T* = 305, 350, and 415°C, WHSV = 1.3 h^–1^, and *P* = 101 kPa (abs).

*T* (°C)	305	350	415
Gases	(mol%)	(mol%)	(mol%)
CO_2_	1.81	16.23	21.27
CO	0.46	2.6	3.59
C1	0.22	0.35	0.99
C2	0	0.58	0.68
C2 ^=^	1.13	2.3	1.45
C3	0.82	3.68	3.33
C3 ^=^	0.96	5.81	15.88
C4	7.1	9.63	11.54
C4 ^=^	2.63	5.66	6.2
Sub-Total	15.13	46.85	64.93
Liquids			
C5	1.26	0.57	0.47
C6	2.33	0.4	0
Benzene	0.32	0.92	1.42
C7	5.78	4.64	3.47
C8	8.85	8.52	7.62
C9	22.19	10.25	4.57
C10	5.53	3.52	2.13
C11	1.63	1.75	1.18
C12	1.99	1.22	1.14
C13	1.94	1.16	1.07
Sub-Total	51.84	32.95	23.09
Water	26.41	14.61	11.98
Acetone	6.62	5.59	0
Total	100	100	100

**Fig 11:**
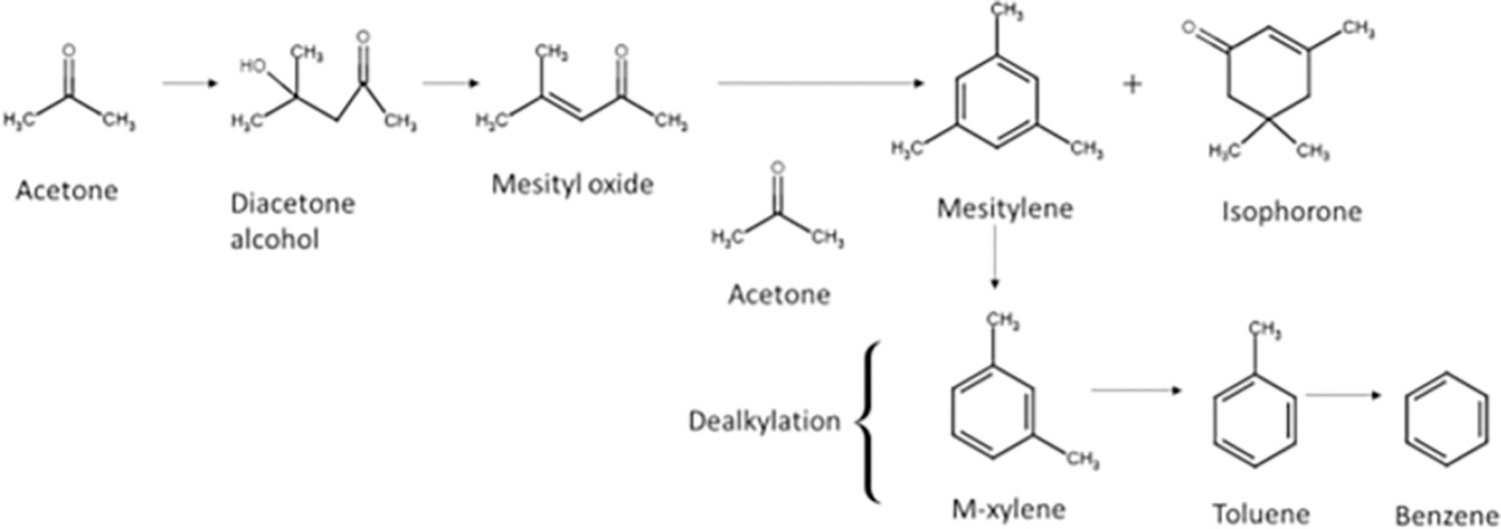
Reaction pathway proposed in the aldol condensation of acetone using HZSM-5.

Fig 11 shows the main reaction pathway observed for the acetone reaction. The catalytic aldo-condensation of acetone is very complex and has numerous products; however, diacetone alcohol, mesityl oxide, phorone, mesitylene, and isophorone are the molecules that were in the products. In this study, the proposed reaction pathway is that mesityl oxide is the main intermediate, which produces mesitylene and then cracks and dealkylates to the aromatics. The same reaction pathway was proposed by Salvapati *et al*. (1989) and Cruz-Cabeza *et al*. (2012). However, Tago *et al*. (2011) showed a different reaction route where isobutylene is the main intermediate, producing aromatics and gaseous hydrocarbons. This mechanism was not observed in the current study [11,12].

### 3.2. Mixed ketone conversion

The following section describes results for mixed ketones. For mixed-ketone reactions, HZSM-5(280) was selected because it did not deactivate for the acetone reaction, and it produced more liquid products than gaseous products.

#### 3.2.1. Catalyst stability

**Fig 12:**
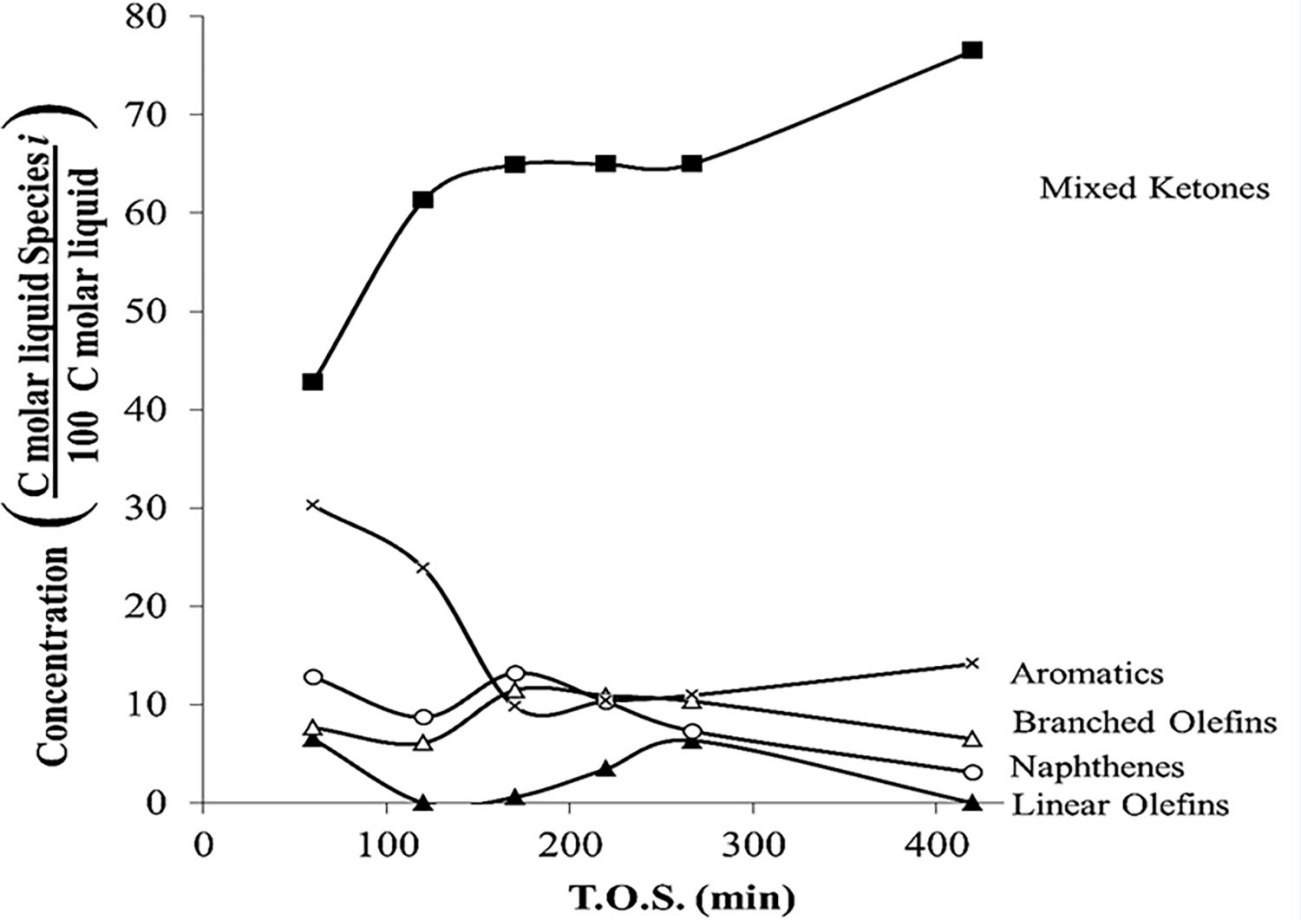
Liquid product distribution for mixed-ketone reaction over HZSM-5(280), WHSV = 1.92 h^–1^, *T =* 510°C, and *P =* 101 kPa (abs).

Fig 12 shows the liquid product distribution over HZSM-5 during T.O.S. at *P* = 101 kPa (abs) and 510°C. During this time, the catalyst also deactivated because the product distribution was affected by changing T.O.S. At very low T.O.S., the amount of unreacted ketones was 42% (70 min); however, at high T.O.S., the unreacted ketones increased to 75% (410 min). On the other hand, the amount of aromatics decreased from 30% (70 min) to 16% (410 min). According to Fuhse and Bandermann (1987), aromatics are the most abundant products for the ketone reaction [10]. Fig 12 shows the amount of aromatics is always higher than the other hydrocarbons. Furthermore, the amount of saturated hydrocarbons (paraffins and isoparaffins) was 0%. According to Chang and Silvestri (1977), the most abundant product for the ketone reaction is aromatics [7]; however, over time branched olefins and naphthenes are produced (Fig 12).

**Fig 13:**
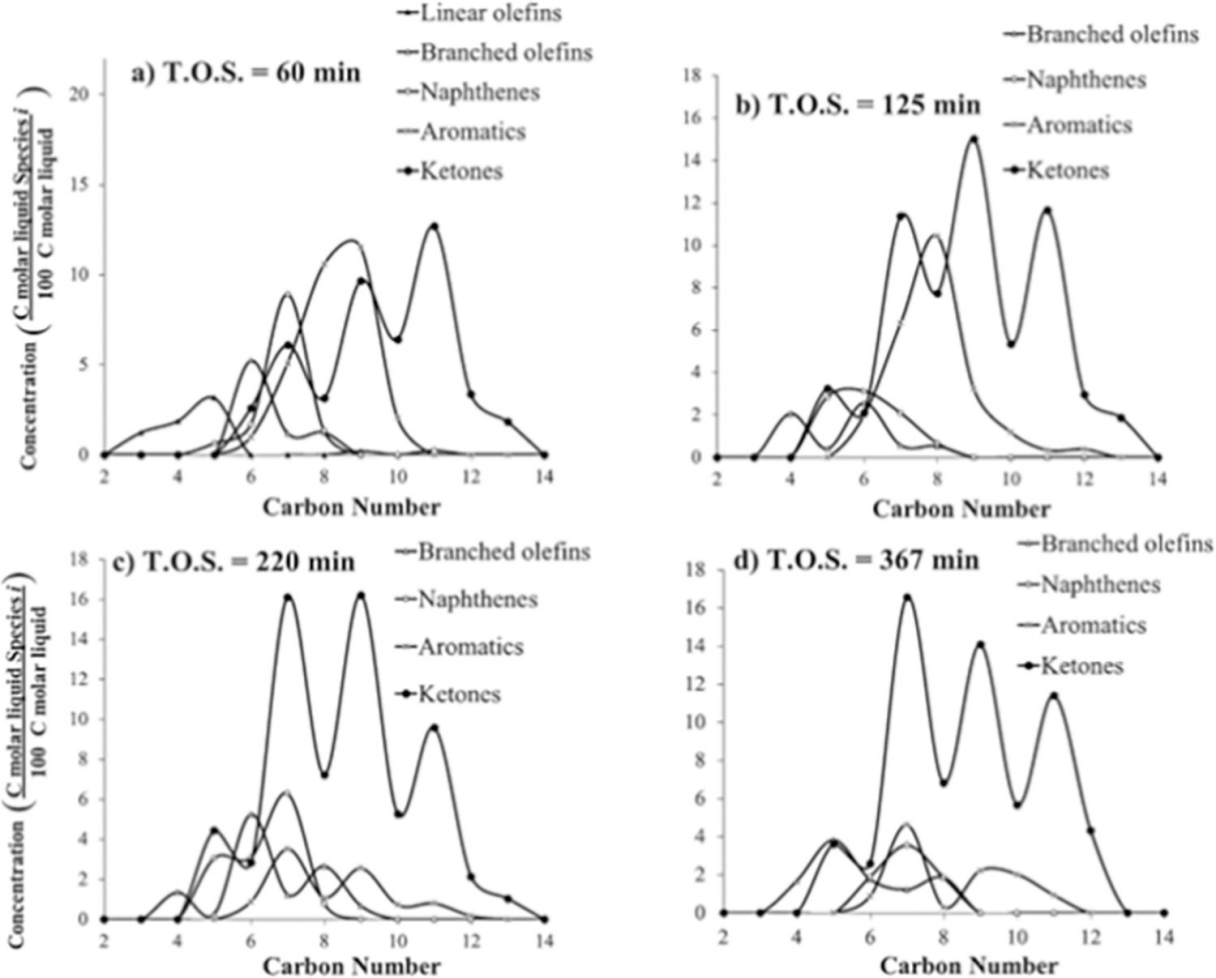
Liquid carbon product distribution of mixed-ketone reaction over HZSM-5(280), WHSV = 1.9 h^–1^, *T* = 590°C, and *P* = 101 kPa (abs).

Fig 13 illustrates the carbon liquid product distribution of mixed ketone over HZSM-5 at different T.O.S., WHSV = 1.9 h^–1^, *T* = 590°C, and *P* = 101 kPa (abs). As T.O.S. increases, conversion and hydrocarbon products decrease. For instance, C9 aromatics decrease from 12% (T.O.S. = 60 min) to 3% (T.O.S. = 367 min). On the other hand, unreacted ketones increase with longer T.O.S. For instance, C7 ketone increases from 6% (T.O.S. = 60 min) to 17% (T.O.S. = 367 min).

Fig 13 illustrates an example of the liquid product of mixed ketone over HZSM-5. Aromatics are centered on C8, ranging from C6 to C12 similar to previous experiments. Naphthenes and branched olefins are centered on C7 and C6, respectively. Naphthene distribution ranges from C6 to C9, and branched olefins range from C5 to C9.

#### 3.2.2. Effect of varying temperature

**Fig 14:**
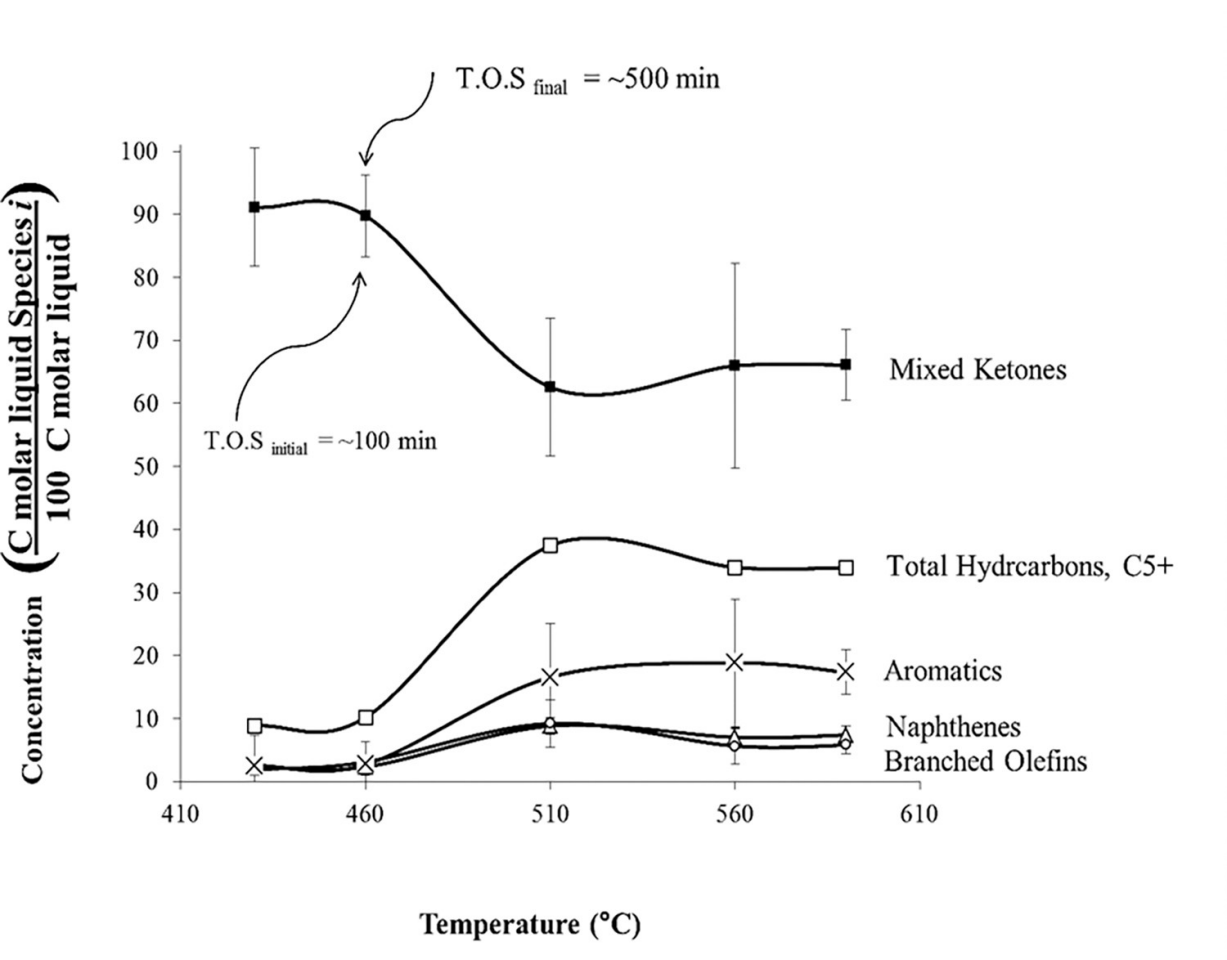
Liquid product distribution for mixed-ketone reaction over HZSM-5(280), WHSV = 1.9 h^–1^, and *P* = 101 kPa (abs). (Error bars are ± 1σ).

Fig 14 illustrates the types of liquid-phase products at different temperatures. At lower temperatures, unconverted ketones are about 90% (420–460°C); however, at higher temperatures, unconverted ketones decrease to 62% (510–590°C). Aromatics are also the most abundant hydrocarbon product (20%). It is noteworthy that the conversion is about the same from 510 to 590°C (~40%), meaning there is no benefit to increasing the temperature to more than 510°C. The maximum amount of hydrocarbon product obtained was 40% at 510°C. Mixed ketone transformation to hydrocarbon is favored at high temperatures. The liquid products from acetone and mixed ketone are mainly aromatics ranging from C6 to C11. At very low temperatures, the mixed ketone conversion was only 9% (420°C); however, at *T* ≥ 510°C, the conversion increased to 41%. The reaction products were aromatics, naphthenes, and branched olefins. Low-molecular-weight (LMW) ketones had higher conversions than high-molecular-weight (HMW) ketones. In all runs above 510°C, acetone conversion is 100%. The concentration of reaction products changed over time because of catalyst coking.

**Fig 15:**
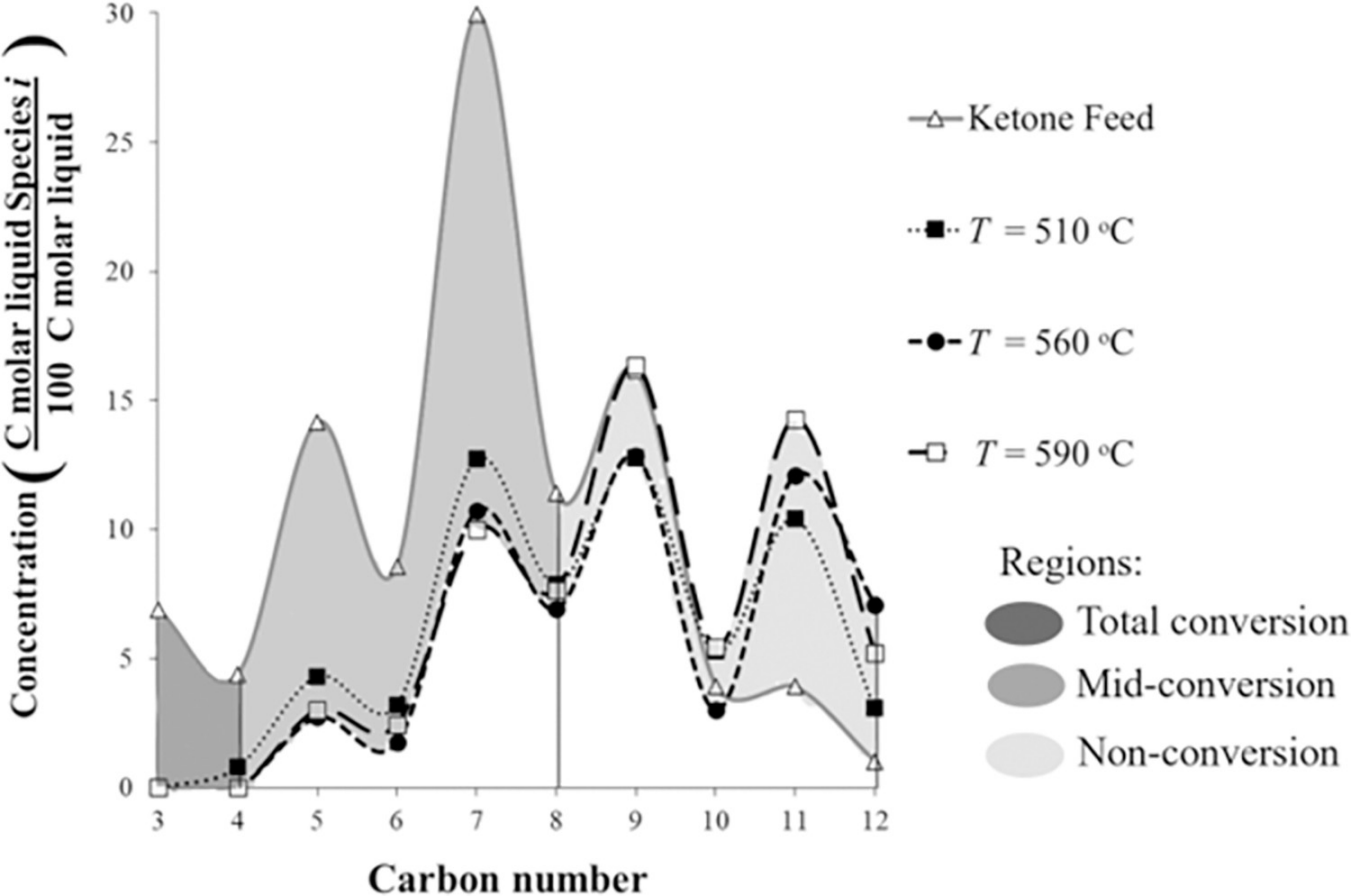
Liquid unreacted product ketone distribution of mixed-ketone reaction over HZSM-5(280), WHSV = 1.9 h^–1^, and *P* = 101 kPa (abs).

For temperatures 510, 560, and 590°C, the conversion is about 40%. For the mixed-ketone reaction, Fig 15 compares the unreacted ketone product distribution with the ketone feed at these temperatures. According to Fuhse and Bandermann (1987), it is expected that acetone, 2-butanone, and 2-pentanone with C/H ratio >0.6 had lower conversion than the remaining ketones (C/H ratio <0.6). However, Fig 15 illustrates that LMW ketones (C3–C7) had higher conversion than HMW ketones (C9–C12). For instance, the acetone feed concentration is 7%; however, the unreacted acetone in the liquid product is 0% (510, 560, and 590°C); therefore, the acetone conversion is 100%. Conversely, 2-heptanone feed concentration is ~29%; however, the unreacted 2-heptanone concentration in the products is ~10%; therefore, the conversion of 2-heptanone is incomplete. On the other hand, HMW ketone has a lower conversion. For instance, 2-nonanone feed concentration is 16%; however, the unreacted 2-nonanone concentration is 14%; therefore, the conversion of 2-nonanone is about 12%. Moreover, 2-decanone does not react at this temperature range; the conversion is 0%.

For mixed ketones, LMW ketones have higher conversion over HZSM-5 than HMW ketones, which might result because small molecules can more easily enter into the channels of HZSM-5 [20]. For instance, acetone size is 0.31 nm and HZSM-5 channel size is 0.51 nm; therefore, acetone not only can react on the surface but also in the internal pores of HZSM-5. On the other hand, larger molecules such as 9-nonene (kinetic diameter >0.5 nm) can only react at the catalyst surface [20]. Unlike Fuhse and Bandermann (1987) [10] where HMW ketones showed higher conversions, our previous work has already mentioned this behavior [4]. In the MixAlco^TM^ process, during the hydrogenation of ketones to hydrocarbons, the conversion of HMW ketones to alcohols was low, therefore LMW and HMW ketones were mixed to achieve better performance. Even skipping the hydrogenation step, which adds expense in the industrial scale-up, the conversion of LMW ketones to hydrocarbons reached 100%, mostly C8-centered aromatics.

According to Wang *et al*. (2008), for proper catalyst performance, the catalyst pore size catalyst relative to the molecule size is very important. The reaction they studied was cracking of 1,3,5-trimethyl benzene (TMB) and 1,2,4-TMB over nanoscale HZM-5 and microscale HZSM-5. The kinetic diameter of 1,3,5-TMB and 1,2,4-TMB is 0.75 and 0.67 nm, respectively, and the catalyst pore opening is 0.5 nm. They concluded that both molecules do not enter the channel of ZSM-5 and only react on the surface [20].

**Fig 16:**
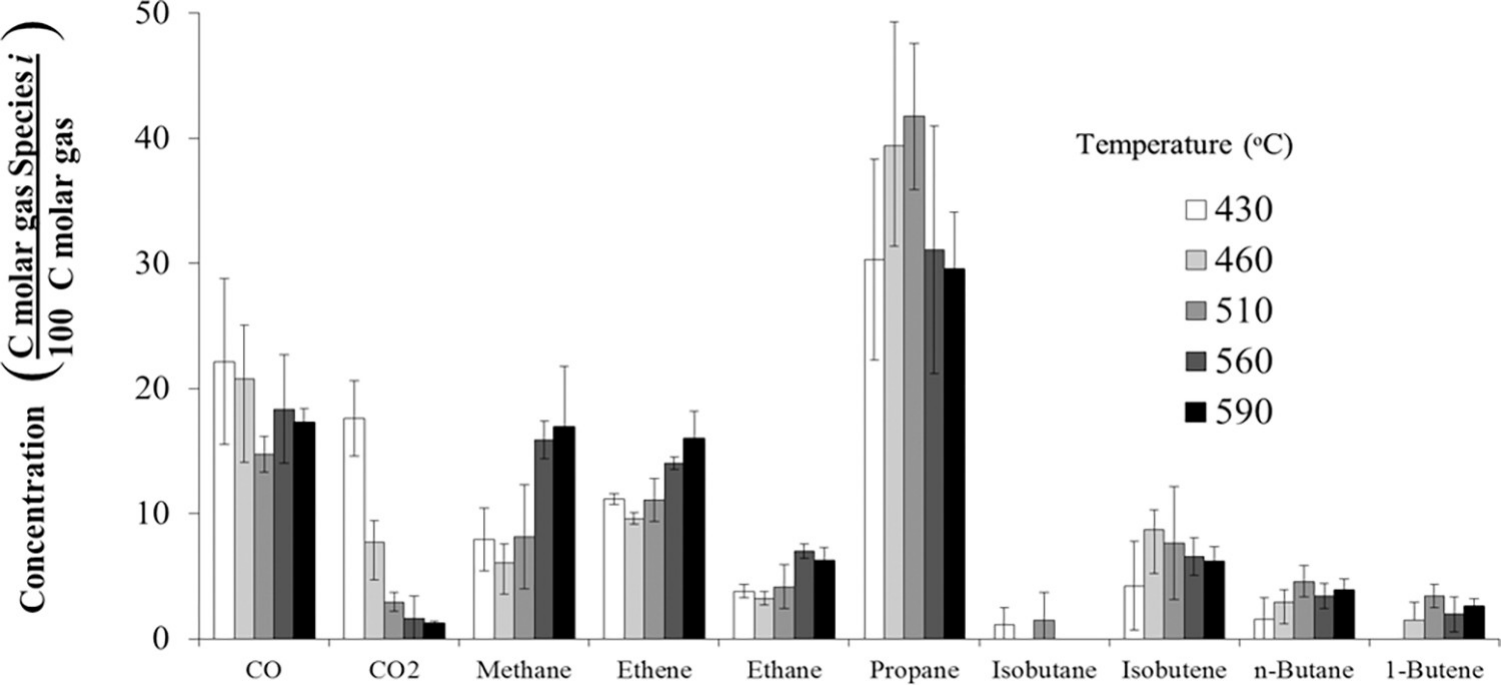
Gaseous product distribution of mixed-ketone reaction over HZSM-5(280), WHSV = 1.9 h^–1^, and *P* = 101 kPa (abs).

Fig 16 shows the gas product distribution of mixed ketone over HZSM-5(280) at different temperatures. Propane is the most abundant gas produced (~40%), similar to the acetone reaction (Fig 4). The amounts of CO and CO_2_ decrease with temperature. At low temperatures, the amount of CO_2_ was 18% (430°C); however, at high temperatures, the amount of CO_2_ decreases to 1% (590°C). On the other hand, methane, ethane, and ethene increase with temperature. For instance, methane was 8% (430°C); however, at high temperatures, methane increased to 18% (590°C). The gaseous products (methane, CO, ethane, ethene, and CO_2_) are similar in composition to the acetone reaction. The gases are mostly saturated with hydrogen, which are difficult to recycle in an oligomerization reactor. Propane is the most abundant product in the gas phase.

Finally, commercial gasoline carbon distribution ranges from C5 to C12, and aromatics are usually 40% of regular commercial gasoline [23]. The octane number of aromatics are over 100, and they usually come from petroleum reforming and are blended with virgin gasoline to improve the octane number. The aromatics produced from acetone and mixed ketones can be blended with virgin gasoline to improve octane number, as has been described previously [24].

The results of the current study must be interpreted in light of several limitations. First, the reported findings may be specific to this commercial catalyst (i.e., HZSM-5). Second, this study focused limited resources on practical engineering and industrialization, rather than theory; therefore, the findings reported herein are applicable primarily for industrial applications, such as the one presented by Taco *et al*. (2014) [4]. Finally, the experiments were conducted on a medium-scale laboratory packed-bed oligomerization rector; however, the findings may be scaled up for industrial applications. At industrial scale, an important design consideration of packed-bed reactors is maintaining the catalyst bed at constant temperature.

## 4. Conclusions

This paper explores the transformation of ketones to hydrocarbons using HZSM-5 catalyst, the last step of the MixAlco™ process. The MixAlco™ process is a version of the carboxylate platform, a leading option for converting lignocellulose to liquid hydrocarbons. Aromatics are the main liquid hydrocarbon products. These high-octane (>100) aromatics from ketones can be blended with low-octane hydrocarbons to increase the octane number of gasoline. Our results indicate that the main hydrocarbon products of acetone and mixed ketone reactions over HZSM-5 are aromatics (liquids) and propane (gases). Aromatics have a Gaussian product distribution centered either on C8 or C9, depending on temperature and WHSV. In this study, under some conditions, acetone conversion was 100%. In contrast, the highest mixed ketones conversion was ~40% because the catalyst rapidly deactivated at the high temperatures (*T* ≥ 510°C) needed to reach higher conversion. In addition, the mixed ketones studied in this paper were produced from a MixAlco^TM^ fermentation operated at 40°C, which favors HMW acids, and hence HMW ketones. This study shows that HMW ketones have very low conversion to hydrocarbons; whereas, acetone with the right conditions has 100% conversion. Increasing the fermentation temperature to 55°C strongly favors acetic acid [4], which results in acetone as the dominant ketone product. Acetone can be transformed to aromatic hydrocarbons with 100% conversion.

Finally, without hydrogen input to the MixAlco^TM^ process, biomass can be converted to high yields of aromatic hydrocarbons from acetone. Future studies on mixed-ketone transformation should be explored with other catalyst options to increase mixed ketone conversion.

## Supporting information

S1 Fig Formation of reaction products in the auto-condensation of acetone. (TIF)

S2 Fig Liquid product distribution of acetone reaction over HZSM-5(80), *T* = 350°C, *P* = 101 kPa (abs). (TIF)

S3 Fig Product distribution of gases and liquids for the acetone reaction over HZSM-5(280), *T* = 415°C, *P* = 101 kPa (abs). (TIF)

S4 Fig Liquid product distribution of acetone reaction over HZSM-5(280), *T* = 415°C, *P* = 101 kPa (abs). (TIF)

S5 Fig Liquid product distribution for mixed ketone reaction over HZSM-5(280), WHSV = 1.92 h^–1^, *T* = 430°C, and *P* = 101 kPa (abs). (TIF)

S1 Table Product distribution of acetone reaction over HZSM-5 catalyst. (DOCX)

S2 Table C/H ratio of the mixed ketone obtained from paper and chicken manure. (DOCX)

S3 Table Compound distribution for the acetone reaction over HZSM-5(280), WHSV = 1.3 h^–1^, *T* = 415°C, and *P* = 790 kPa (abs). (DOCX)

S4 Table Compound distribution for the acetone reaction over HZSM-5(280), WHSV = 3.9 h^–1^, *T* = 415°C, and *P* = 790 kPa (abs). (DOCX)

S5 Table Compound distribution for the acetone reaction over HZSM-5(280), WHSV = 5.2 h^–1^, *T* = 415°C, and *P* = 790 kPa (abs). (DOCX)
