# Supplementary figures and images for: Conversion of acetone and mixed ketones to hydrocarbons using HZSM-5 catalyst in the carboxylate platform
Source: PLoS One. 2022 Nov 21;17(11):e0277184. doi: 10.1371/journal.pone.0277184 (PMC9678301; doi:10.1371/journal.pone.0277184)

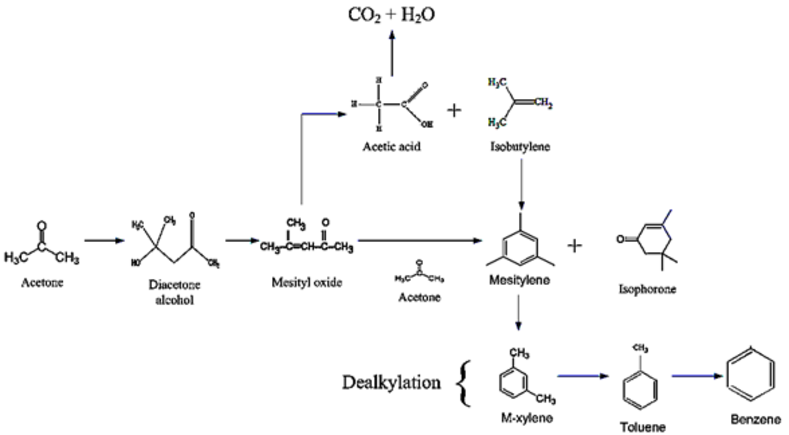

Supplement: S1 Fig — (TIF) [file pone.0277184.s001.tif]

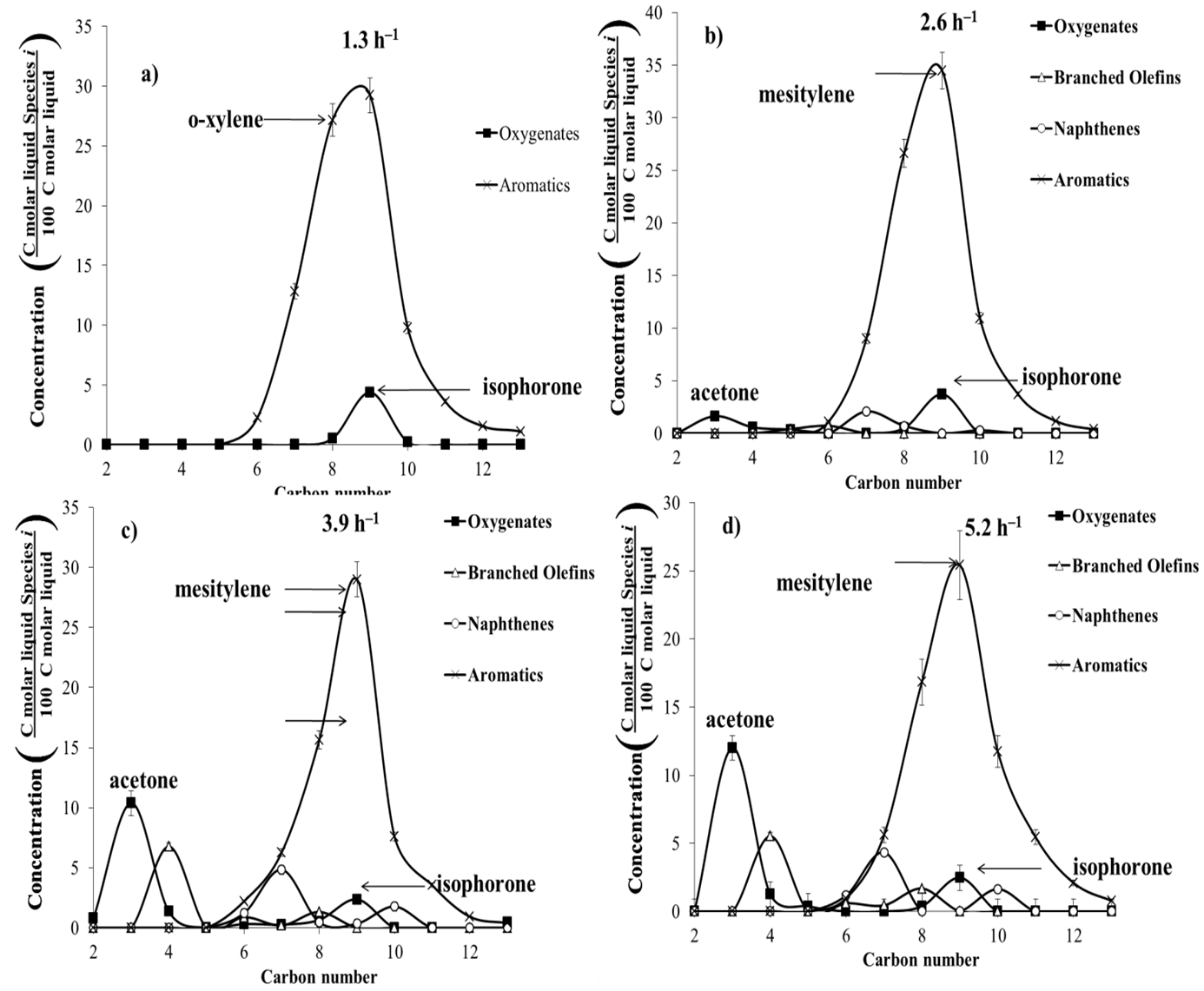

Supplement: S2 Fig — (TIF) [file pone.0277184.s002.tif]

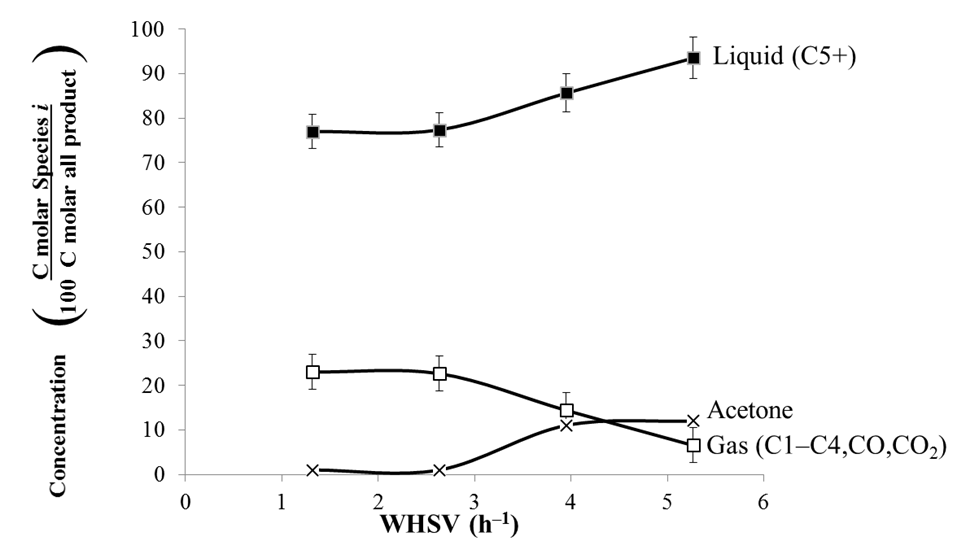

Supplement: S3 Fig — (TIF) [file pone.0277184.s003.tif]

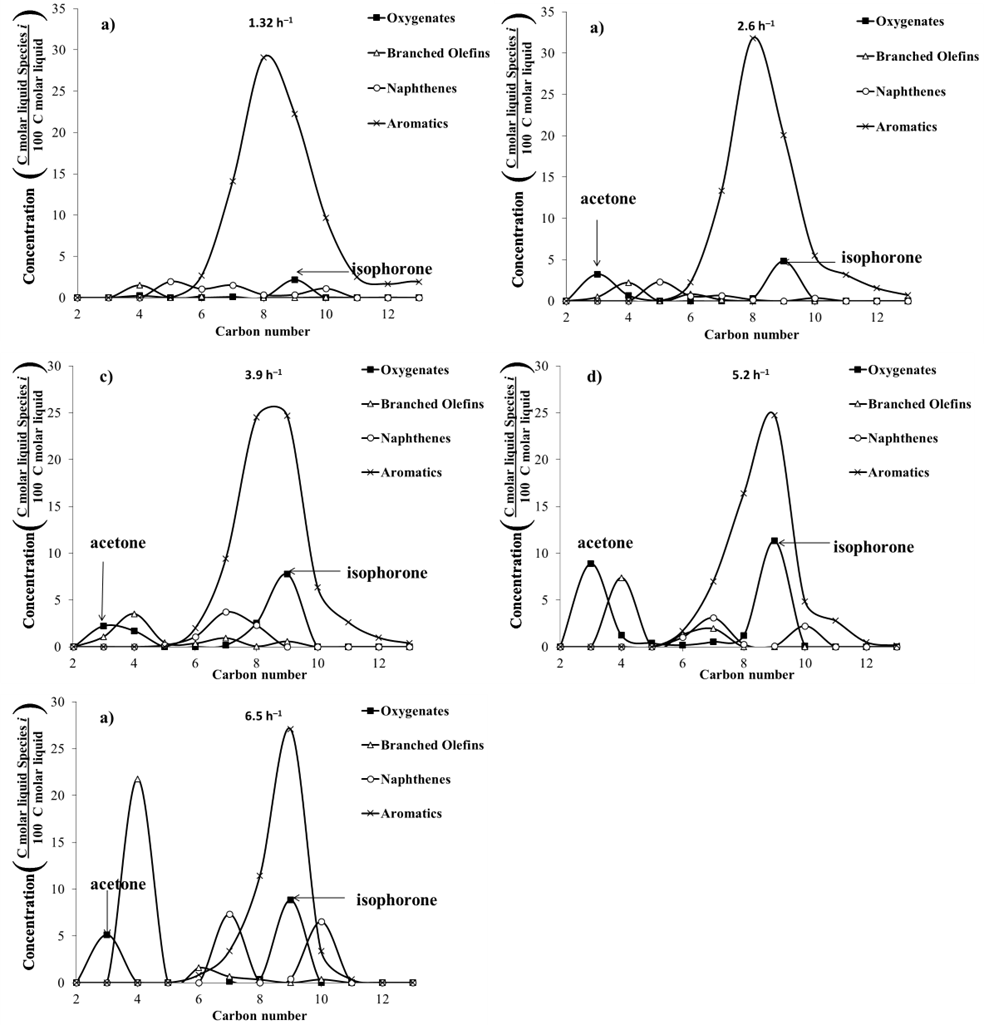

Supplement: S4 Fig — (TIF) [file pone.0277184.s004.tif]

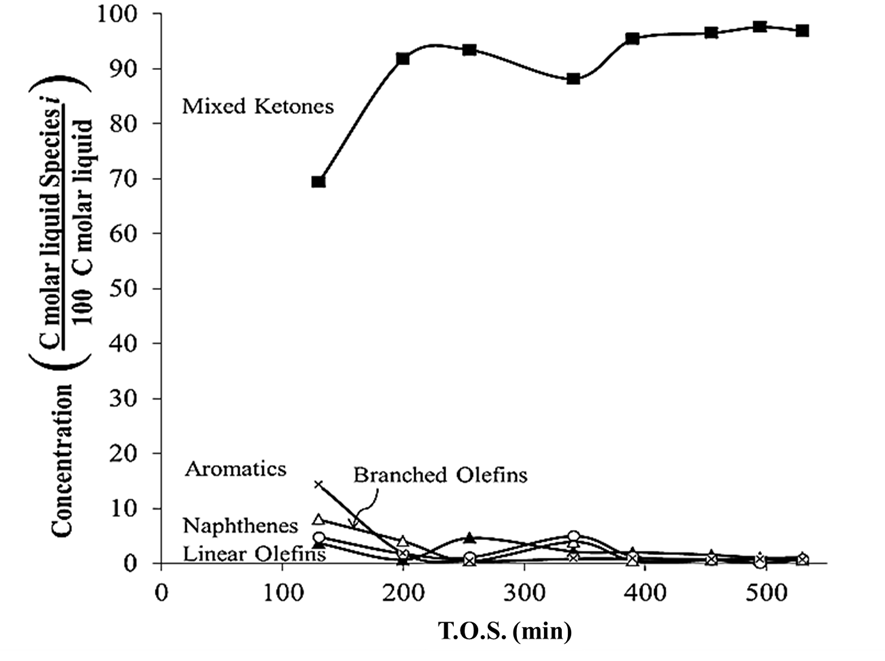

Supplement: S5 Fig — (TIF) [file pone.0277184.s005.tif]
