# Supplementary material for: Conversion of acetone and mixed ketones to hydrocarbons using HZSM-5 catalyst in the carboxylate platform
Source: PLoS One. 2022 Nov 21;17(11):e0277184. doi: 10.1371/journal.pone.0277184 (PMC9678301; doi:10.1371/journal.pone.0277184)
Supplement: S1 Table — (DOCX) [file pone.0277184.s006.docx]

Table 1. Product distribution of acetone reaction over HZSM-5 catalyst [5]

| Reaction Conditions |  |  |  |  |
| --- | --- | --- | --- | --- |
| *T* (°C) | 250 | 288 | 329 | 399 |
| WHSV (h^–1^) | 8.0 | 8.0 | 8.0 | 8.0 |
| Conversion (%) | 3.9 | 6.0 | 24.5 | 95.3 |
| Carbon Selectivity (%) |  |  |  |  |
| Diacetone alcohol | 3.5 | 2.9 | 0.1 | – |
| Mesityl oxide | 27.3 | 19.7 | 1.2 | – |
| Isophorone | – | <0.1 | 5.3 | – |
| Other O-compounds | 6.0 | 15.0 | <0.1 | – |
| CO + CO_2_ | – | 0.7 | 10.0 | 6.1 |
| Hydrocarbons | 63.2 | 61.2 | 83.4 | 93.9 |
| Hydrocarbon Distribution (wt%) |  |  |  |  |
| Methane | – | – | 0.2 | 0.1 |
| Ethane | – | – | 0.4 | 0.2 |
| Ethylene | <0.1 | <0.1 | 1.2 | 2.4 |
| Propane |  | 0.3 | 1.9 | 4.2 |
| Propylene | 2.5 | 3.8 | 4.2 | 5.2 |
| *i*-Butane | – | – | 0.1 | 3.9 |
| *n*-Butane | – | – | – | 1.7 |
| *i*-Butene | 19.1 | 31.3 | 83.3 | 3.6 |
| *n*-Butene | – | – | <0.1 | 2.3 |
| *i*-Pentane | – | – | – | 1.5 |
| *n*-Pentane | – | – | – | 0.6 |
| Pentenes | – | – | – | 2.5 |
| C6+ Aliphatics | 19.1 | 3.8 | 1.6 | 8.2 |
| Benzene | – | – | – | 2.6 |
| Toluene | – | – | 0.1 | 13.0 |
| Ethylbenzene | – | – | – | 2.7 |
| Xylenes | – | 1.3 | 2.1 | 22.3 |
| 1,2,3-Trimethylbenzene | – | <0.1 | <0.1 | 1.1 |
| 1,2,4-Trimethylbenzene | – | 7.0 | 2.0 | 8.8 |
| 1,3,5-Trimethylbenzene | 59.3 | 52.5 | 2.6 | 0.6 |
| Other C9 Aromatics | – | – | 0.3 | 9.7 |
