# Supplementary material for: Conversion of acetone and mixed ketones to hydrocarbons using HZSM-5 catalyst in the carboxylate platform
Source: PLoS One. 2022 Nov 21;17(11):e0277184. doi: 10.1371/journal.pone.0277184 (PMC9678301; doi:10.1371/journal.pone.0277184)
Supplement: S2 Table — (DOCX) [file pone.0277184.s007.docx]

**Table 2.** C/H ratio of the mixed ketone obtained from paper and chicken manure.

| Mixed Ketones | C/H (mol/mol) |
| --- | --- |
|  |  |
| Acetone | 0.75 |
| 2-Butanone | 0.67 |
| 2-Pentanone | 0.63 |
| 2-Hexanone | 0.60 |
| 2-Heptanone | 0.58 |
| 2-Octanone | 0.57 |
| 4-Nonanone | 0.56 |
| 5-Decanone | 0.56 |
| 6-Undecanone | 0.55 |
| 6-Dodecanone | 0.55 |
| 6-Tridecanone | 0.54 |
