# Supplementary material for: Conversion of acetone and mixed ketones to hydrocarbons using HZSM-5 catalyst in the carboxylate platform
Source: PLoS One. 2022 Nov 21;17(11):e0277184. doi: 10.1371/journal.pone.0277184 (PMC9678301; doi:10.1371/journal.pone.0277184)
Supplement: S3 Table — (DOCX) [file pone.0277184.s008.docx]

**Table 3.** Compound distribution for the acetone reaction over HZSM-5(280), WHSV = 1.3 h^–1^, *T* = 415 °C, and *P* = 790 kPa (abs).

| Conc% | Name | Type | Carbon # |
| --- | --- | --- | --- |
| 20.57 | Benzene, 1,3,5-trimethyl- | A | 9 |
| 19.9 | Benzene, 1,4-dimethyl- | A | 8 |
| 13.82 | Benzene, methyl- | A | 7 |
| 5.72 | Benzene, 1,2,3-trimethyl- | A | 9 |
| 5.7 | Benzene, 1,3-dimethyl- | A | 7 |
| 5.32 | Benzene, 1,2,3,5-tetramethyl- | A | 10 |
| 3.97 | 2-Propanone | OXI | 3 |
| 3.11 | Benzene | A | 6 |
| 2.42 | 1-Propene, 2-methyl- | O | 4 |
| 1.38 | Benzene, 1-ethyl-4-methyl- | A | 9 |
| 1.24 | Benzene, ethyl- | A | 8 |
| 1.15 | Naphthalene, 1-methyl- | A | 11 |
| 0.9 | Benzene, 1,2,4-trimethyl- | A | 9 |
| 0.86 | 1H-Indene, 2,3-dihydro-5-methyl- | A | 10 |
| 0.84 | Benzene, 1-methyl-3-(1-methylethyl) | A | 10 |
| 0.84 | Naphthalene, 1,6-dimethyl- | A | 12 |
| 0.55 | Benzene, 1-methyl-3-(1-methylethyl) | A | 10 |
| 0.54 | 1H-Indene, 2,3-dihydro-4,7-dimethyl | A | 11 |
| 0.46 | Pentane, 2-methyl- | I | 6 |
| 0.41 | 2-Butanone | OXI | 4 |
| 0.41 | 1H-Indene, 2,3-dihydro- | A | 9 |
| 0.41 | M-TOLYL-DIMETHYLACETALDEHYDE | A | 12 |
| 0.36 | P-MENTHA-1,5,8-TRIENE | A | 10 |
| 0.35 | 1,3-Hexadiene, 3-ethyl-2,5-dimethyl | O | 10 |
| 0.34 | Phenol, 2,4-dimethyl- | OXI | 8 |
| 0.34 | Cyclohexanone, 3,3,5-trimethyl- | OXI | 9 |
| 0.34 | Benzene, 1,4-dimethyl-2-(2-methylpr | A | 12 |
| 0.32 | Benzene, 1-ethyl-2,4-dimethyl- | A | 10 |
| 0.3 | 2,2-Dimethyl-1-isopropenyl-cyclopen | N | 10 |
| 0.3 | 1-Butyl-2,3,6-trimethylbenzene | A | 13 |
| 0.29 | 3-Penten-2-one, 4-methyl- | OXI | 6 |
| 0.28 | Cyclopentane, methyl- | N | 6 |
| 0.28 | Benzene, 1-methyl-2-propyl- | A | 10 |
| 0.27 | Benzene, 1-ethyl-3,5-dimethyl- | A | 10 |
| 0.27 | 1,3,4,6,7,11b-Hexahydro-2H-benzo[b] | A | 12 |
| 0.26 | Benzene, 1,3-diethyl- | A | 10 |
| 0.26 | 1-Butyl-2,3,6-trimethylbenzene | A | 13 |
| 0.25 | Cyclopentene, 4,4-dimethyl- | NO | 7 |
| 0.23 | Benzene, 1-ethyl-3-methyl- | A | 9 |
| 0.23 | Benzene, (3-methyl-2-butenyl)- | A | 11 |
| 0.23 | Naphthalene, 2,3-dimethyl- | A | 12 |
| 0.23 | Naphthalene, 2-(1-methylethyl)- | A | 13 |
| 0.22 | Naphthalene, 1,4,6-trimethyl- | A | 13 |
| 0.21 | Naphthalene, 1,4,6-trimethyl- | A | 13 |
| 0.2 | Cyclopentane, 1,2-dimethyl-, cis- | N | 7 |
| 0.2 | 1H-Indene, 2,3-dihydro-4,7-dimethyl | A | 11 |
| 0.2 | Naphthalene, 1-ethyl- | A | 12 |
| 0.19 | Benzene, 1-ethyl-3,5-dimethyl- | A | 10 |
| 0.18 | Phenol, 2,4,6-trimethyl- | OXI | 9 |
| 0.18 | Cyclohexene, 1-methyl- | NO | 7 |
| 0.18 | Pentane, 3-methyl- | I | 6 |
| 0.18 | Naphthalene, 1,2,3,4-tetrahydro-2,2 | A | 12 |
| 0.17 | 1,3-Cyclohexadiene, 1,5,5,6-tetrame | NO | 10 |
| 0.17 | Benzene, 1,2,4-trimethyl-5-(1-methy | A | 10 |
| 0.17 | 1H-Indene, 2,3-dihydro-4,7-dimethyl | A | 11 |
| 0.16 | Benzene, 2,4-dimethyl-1-(1-methylet | A | 11 |
| 0.16 | Naphthalene, 1,4,6-trimethyl- | A | 13 |
| 0.15 | 4-Ethylindan | A | 11 |
| 0.14 | 3-Ethoxy-4-ethylthiocyclobutenedion |  |  |
| 0.13 | Benzene, pentamethyl- | A | 11 |
| 0.12 | Benzene, 1-ethyl-4-(1-methylethyl)- | A | 11 |
| 0.12 | 1,2-DIHYDRO-4-ETHYL-5-METHYLPYRROLO | A | 13 |
| 0.11 | .BETA.-OCIMENE-X | U | 8 |
| 0.11 | Naphthalene, 1,2,3,4-tetrahydro-2-m | A | 10 |
| 0.1 | Naphthalene, 1,2,3,4-tetrahydro-2,7 | A | 10 |
