# Supplementary material for: Conversion of acetone and mixed ketones to hydrocarbons using HZSM-5 catalyst in the carboxylate platform
Source: PLoS One. 2022 Nov 21;17(11):e0277184. doi: 10.1371/journal.pone.0277184 (PMC9678301; doi:10.1371/journal.pone.0277184)
Supplement: S4 Table — (DOCX) [file pone.0277184.s009.docx]

**Table 4.** Compound distribution for the acetone reaction over HZSM-5(280), WHSV = 3.9 h^–1^, *T* = 415 °C, and *P* = 790 kPa (abs).

| Conc% | Name | Type | Carbon # |
| --- | --- | --- | --- |
| 16.72 | Benzene, 1,3-dimethyl- | A | 8 |
| 10.3 | Benzene, methyl- | A | 7 |
| 10.25 | Benzene, 1-ethyl-3-methyl- | A | 9 |
| 9.16 | Benzene, 1,2,3-trimethyl- | A | 9 |
| 6.94 | Benzene, 1,2,3-trimethyl- | A | 9 |
| 4.46 | 2-Propanone | OXI | 3 |
| 4.08 | Benzene, 1,4-dimethyl- | A | 8 |
| 3.9 | 2-Cyclohexen-1-one, 3,5,5-trimethyl | OXI | 9 |
| 2.97 | Benzene, ethyl- | A | 8 |
| 2.57 | Benzene, 1,2,3,5-tetramethyl- | A | 10 |
| 2.07 | 1-Propene, 2-methyl- | O | 3 |
| 1.51 | Cyclohexanone, 3,3,5-trimethyl- | OXI | 9 |
| 1.41 | Benzene | A | 6 |
| 1.24 | Benzene, 1-methyl-3-(1-methylethyl) | A | 10 |
| 1.2 | Benzene, 1,4-diethyl- | A | 10 |
| 1.13 | 1H-Indene, 2,3-dihydro-5-methyl- | A | 10 |
| 0.87 | Phenol, 2,5-dimethyl- | OXI | 8 |
| 0.84 | 1-Butene, 2-methyl- | O | 5 |
| 0.84 | 1H-Indene, 2,3-dihydro-4,7-dimethyl | A | 11 |
| 0.81 | Naphthalene, 1-methyl- | A | 11 |
| 0.78 | Benzene, 1,2,4,5-tetramethyl- | A | 10 |
| 0.78 | Naphthalene, 1,5-dimethyl- | A | 12 |
| 0.65 | 1,3-Cyclohexadiene, 1,5,5,6-tetrame | NO | 10 |
| 0.63 | Benzene, 1,3-diethyl- | A | 10 |
| 0.56 | Benzene, 1-methyl-3-propyl- | A | 10 |
| 0.55 | Cyclopentene, 4,4-dimethyl- | NO | 7 |
| 0.53 | 2-Methyl-4(5)-tert-butylimidazole | U | 8 |
| 0.52 | .alpha.-Terpinene | U | 10 |
| 0.47 | Benzene, 1-ethyl-4-methyl- | A | 9 |
| 0.45 | 1-Butene, 2,3-dimethyl- | O | 6 |
| 0.43 | Phenol, 2,4,6-trimethyl- | OXI | 9 |
| 0.43 | Cyclopentene, 1,5-dimethyl- | NO | 7 |
| 0.42 | 2-Butanone | OXI | 4 |
| 0.42 | Benzene, propyl- | A | 9 |
| 0.4 | Pentane, 2-methyl- | I | 6 |
| 0.36 | 1-Pentene, 3,4-dimethyl- | I | 7 |
| 0.35 | Benzene, 1-(2-butenyl)-2,3-dimethyl | A | 12 |
| 0.3 | 1-Butyl-2,3,6-trimethylbenzene | A | 13 |
| 0.29 | Benzene, 1,2,4,5-tetramethyl- | A | 10 |
| 0.29 | 1H-Indene, 2,3-dihydro-4,7-dimethyl | A | 11 |
| 0.28 | Cyclopentane, methyl- | N | 6 |
| 0.27 | Benzene, 1,1'-(1-ethenyl-1,3-propan | A | 11 |
| 0.25 | Cyclopentene, 1-methyl- | NO | 6 |
| 0.25 | Naphthalene, 1,2-dihydro-2-methyl- | A | 11 |
| 0.24 | Naphthalene, 2-(1-methylethyl)- | A | 13 |
| 0.23 | 1-Butyl-2,3,6-trimethylbenzene | A | 13 |
| 0.21 | 1(2H)-Naphthalenone, 3,4-dihydro- | OXI | 10 |
| 0.21 | 5-ISOBUTYL-1,2,3,4-TETRAHYDRONAPHTH | A | 12 |
| 0.2 | Cyclohexene, 4-methyl- | NO | 7 |
| 0.2 | 4-Ethylindan | A | 11 |
| 0.19 | Benzene, 1-methyl-4-(1-methylethyl) | A | 10 |
| 0.17 | 1,4-Cyclohexadiene, 3,3,6,6-tetrame | NO | 10 |
| 0.16 | 2-Pentene, 3-methyl-, (Z)- | O | 6 |
| 0.16 | 1,4-Pentadiene, 2,3,3-trimethyl- | O | 8 |
| 0.16 | 1H-Indene, 2,3-dihydro-1-methyl- | A | 10 |
| 0.15 | Phenol, 3,4,5-trimethyl- | OXI | 9 |
| 0.15 | 1,4-Pentadiene, 3,3-dimethyl- | NO | 7 |
| 0.15 | Benzene, (1-methylbutyl)- | A | 11 |
| 0.14 | 1-Methyl-2-(3-pyridyl)azetidine | U | 10 |
| 0.13 | 2-Cyclohexen-1-one, 3,6-dimethyl-6- | OXI | 9 |
| 0.13 | Cyclopentane, 1,2-dimethyl- | N | 7 |
| 0.13 | Hexane, 3-methyl- | I | 7 |
| 0.13 | Benzene, 1-methyl-4-(1-methylethyl) | A | 10 |
| 0.13 | Naphthalene, 1,4,6-trimethyl- | A | 13 |
| 0.12 | Cyclopentane, 1,2-dimethyl- | N | 7 |
| 0.12 | 1H-Indene, 2,3-dihydro-4,5,7-trimet | A | 12 |
| 0.12 | Benzene, 1,3,5-trimethyl-2-(3-methy | A | 12 |
| 0.11 | [1,1'-Biphenyl]-4-amine, 4'-fluoro- | U | 12 |
| 0.11 | 1,4-Pentadiene, 2,3,3-trimethyl- | O | 8 |
| 0.11 | 1,3-CYCLOPENTADIENE, 5-TERT-BUTYL- | NO | 9 |
| 0.11 | Pentane, 3-methyl- | I | 6 |
| 0.11 | Benzene, (1-methyl-2-cyclopropen-1- | A | 10 |
| 0.11 | Benzene, pentamethyl- | A | 11 |
| 0.1 | Benzene, (1-methylethyl)- | A | 9 |
| 0.09 | 1H-Pyrido[3,4-b]indole, 2,3,4,9-tet | U | 12 |
| 0.09 | Phenol, 3-methyl- | OXI | 9 |
| 0.09 | 2,4-Hexadiene, 2,5-dimethyl- | O | 8 |
| 0.09 | Cyclohexene, 1,2-dimethyl- | NO | 8 |
| 0.09 | Cyclopentane, 1-ethyl-3-methyl- | N | 8 |
| 0.09 | Benzene, 1-(2-butenyl)-2,3-dimethyl | A | 12 |
| 0.09 | Naphthalene, 2-ethyl- | A | 12 |
| 0.09 | 1,2-DIHYDRO-4-ETHYL-5-METHYLPYRROLO | A | 13 |
