# Supplementary material for: Conversion of acetone and mixed ketones to hydrocarbons using HZSM-5 catalyst in the carboxylate platform
Source: PLoS One. 2022 Nov 21;17(11):e0277184. doi: 10.1371/journal.pone.0277184 (PMC9678301; doi:10.1371/journal.pone.0277184)
Supplement: S5 Table — (DOCX) [file pone.0277184.s010.docx]

**Table 5.** Compound distribution for the acetone reaction over HZSM-5(280), WHSV = 5.2 h^–1^, *T* = 415 °C, and *P* = 790 kPa (abs).

| Conc% | Name | Type | Carbon # |
| --- | --- | --- | --- |
| 11 | Benzene, 1,4-dimethyl- | A | 8 |
| 10.06 | Benzene, 1-ethyl-4-methyl- | A | 9 |
| 9.64 | Benzene, 1,2,3-trimethyl- | A | 9 |
| 6.72 | Benzene, 1,3,5-trimethyl- | A | 9 |
| 5.94 | 1-Propene, 2-methyl- | O | 5 |
| 5.16 | Benzene, methyl- | A | 7 |
| 4.84 | 2-Cyclohexen-1-one, 3,5,5-trimethyl | OXI | 9 |
| 2.78 | 2-Propanone | OXI | 3 |
| 2.32 | Benzene, 1-methyl-3-(1-methylethyl) | A | 10 |
| 2.29 | Benzene, 1,2,3,5-tetramethyl- | A | 10 |
| 2.25 | CYCLOBUTANE, ISOPROPYLIDEN- | N | 7 |
| 2.12 | Benzene, 1,2-dimethyl- | A | 8 |
| 1.89 | Benzene, 1,2-diethyl- | A | 10 |
| 1.54 | 2-Propanone | OXI | 3 |
| 1.53 | Cyclopentene, 1,5-dimethyl- | NO | 7 |
| 1.48 | 1,3-Cyclohexadiene, 1,2,6,6-tetrame | NO | 10 |
| 1.47 | Benzene, ethyl- | A | 8 |
| 1.46 | 3-Penten-2-one, 4-methyl- | OXI | 6 |
| 1.44 | 2-Butanone | OXI | 4 |
| 1.35 | Isoterpinolene | NO | 10 |
| 1.29 | Benzene, 1-methyl-3-propyl- | A | 10 |
| 1.09 | Benzene | A | 6 |
| 0.94 | Benzene, 1,2,3,5-tetramethyl- | A | 10 |
| 0.88 | 1,3,5-Hexatriene, 3-methyl-, (Z)- | O | 7 |
| 0.86 | 1H-Indene, 2,3-dihydro-4,7-dimethyl | A | 11 |
| 0.69 | TRISPIRO(2.0.2.0.2.1)DECANE | A | 11 |
| 0.6 | Cyclohexanone, 3,3,5-trimethyl- | OXI | 9 |
| 0.56 | Cyclopentene, 1,5-dimethyl- | NO | 7 |
| 0.55 | 1-Butyl-2,3,6-trimethylbenzene | A | 13 |
| 0.54 | Benzene, 1-methyl-2-(1-methylethyl) | A | 10 |
| 0.52 | Cyclopentene, 1-methyl- | O | 6 |
| 0.52 | Naphthalene, 1-methyl- | A | 11 |
| 0.51 | 3,3-Dimethyl-2-isopropyl-cyclopente | NO | 10 |
| 0.5 | 1H-Indene, 2,3-dihydro-5-methyl- | A | 10 |
| 0.49 | Benzene, propyl- | A | 9 |
| 0.48 | 1,3-Cyclohexadiene, 1,2,6,6-tetrame | NO | 10 |
| 0.45 | CYCLOHEXENE, 1,3-DIMETHYL- | NO | 8 |
| 0.4 | 2,4,6-OCTATRIENE, ALL-TRANS | O | 8 |
| 0.4 | Naphthalene, 1,6-dimethyl- | A | 13 |
| 0.39 | Acetic acid | OXI | 2 |
| 0.39 | 1H-Indene, 1,1-dimethyl- | A | 11 |
| 0.38 | Benzene, 1,2,3-trimethyl- | A | 9 |
| 0.35 | Benzene, (1-methylethyl)- | A | 9 |
| 0.35 | Benzene, 1-(2-butenyl)-2,3-dimethyl | A | 12 |
| 0.34 | Cyclohexane, methyl- | N | 7 |
| 0.34 | 1H-Indene, 2,3-dihydro-4,7-dimethyl | A | 11 |
| 0.33 | 2-Pentene, 2-methyl- | O | 6 |
| 0.33 | Benzene, 1-methyl-3-(1-methylethyl) | A | 10 |
| 0.3 | Benzene, (1,2-dimethylpropyl)- | A | 10 |
| 0.3 | Benzene, (1,1-dimethylpropyl)- | A | 11 |
| 0.29 | 1,4-Pentadiene, 2,3,3-trimethyl- | O | 8 |
| 0.29 | Cyclohexane, 1-methyl-4-methylene- | N | 8 |
| 0.27 | Benzene, (1,1-dimethylpropyl)- | A | 11 |
| 0.27 | 1-Butyl-2,3,6-trimethylbenzene | A | 13 |
| 0.26 | 1-Pentene, 3-ethyl- | O | 7 |
| 0.26 | 1H-Indene, 2,3-dihydro- | A | 9 |
| 0.25 | 2-Pentene, 3-methyl-, (E)- | O | 6 |
| 0.24 | Phenol, 2,3,5-trimethyl- | OXI | 9 |
| 0.21 | 1H-Indene, 2,3-dihydro-4,5,7-trimet | A | 12 |
| 0.2 | 2-Hexene, 2,5-dimethyl- | O | 8 |
| 0.19 | 2-Hexanone, 5-methyl- | OXI | 7 |
| 0.19 | 2-Hexene, 2-methyl- | NO | 7 |
| 0.19 | 1H-Indene, 2,3-dihydro-1,6-dimethyl | A | 11 |
| 0.17 | Benzene, 4-(2-butenyl)-1,2-dimethyl | A | 12 |
| 0.16 | 2-Butene, 2,3-dimethyl- | O | 6 |
| 0.15 | 2-Cyclohexen-1-one, 3,6-dimethyl-6- | U | 13 |
| 0.15 | Phenol, 3,4-dimethyl- | OXI | 8 |
| 0.14 | 1-Pentene, 2-methyl- | O | 6 |
| 0.13 | Heptane, 4-methylene- | I | 8 |
| 0.12 | Benzenemethanol, .alpha.-ethyl- | U | 9 |
| 0.12 | Benzene, 1-methyl-4-(1-methylethyl) | A | 10 |
| 0.11 | Phenol, 3-methyl- | OXI | 7 |
| 0.11 | CYCLOPENTANE, 1,2-DIMETHYL-3-METHYL | N | 9 |
| 0.11 | 4-Ethylindan | A | 11 |
| 0.1 | 2-Propanone, 1-(3,5,5-trimethyl-2-c | U | 13 |
| 0.1 | 1,4-Pentadiene, 2,3,3-trimethyl- | O | 8 |
| 0.1 | Cyclohexane, (1-methylethylidene)- | N | 9 |
| 0.1 | .ALPHA.-PINENE, (-)- | A | 9 |
| 0.1 | Benzene, pentamethyl- | A | 11 |
| 0.09 | Cyclopentane, ethylidene- | N | 7 |
| 0.09 | Benzene, diethylmethyl- | A | 11 |
| 0.08 | CYCLONONYNE | NO | 9 |
| 0.08 | 1H-Indene, 2,3-dihydro-1,4,7-trimet | A | 12 |
| 0.07 | N,N'-BIS(2,6-DIMETHYL-6-NITROSOHEPT | U | 8 |
| 0.07 | 1,4-Hexadiene, 3-ethyl- | O | 8 |
| 0.07 | 1,4-Hexadiene, 3-ethyl- | O | 8 |
| 0.07 | Benzene, 1,3,5-trimethyl-2-(3-methy | A | 13 |
| 0.07 | Naphthalene, 2-(1-methylethyl)- | A | 13 |
| 0.06 | 2,5-Heptadiene, (E,E)- | O | 7 |
| 0.06 | Benzene, 1,4-dimethyl-2-(2-methylpr | A | 12 |
| 0.06 | Benzene, (1-cyclohexylethyl)- | A | 14 |
